# Carriage of *Mycoplasma pneumoniae* in the Upper Respiratory Tract of Symptomatic and Asymptomatic Children: An Observational Study

**DOI:** 10.1371/journal.pmed.1001444

**Published:** 2013-05-14

**Authors:** Emiel B. M. Spuesens, Pieter L. A. Fraaij, Eline G. Visser, Theo Hoogenboezem, Wim C. J. Hop, Léon N. A. van Adrichem, Frank Weber, Henriette A. Moll, Berth Broekman, Marjolein Y. Berger, Tineke van Rijsoort-Vos, Alex van Belkum, Martin Schutten, Suzan D. Pas, Albert D. M. E. Osterhaus, Nico G. Hartwig, Cornelis Vink, Annemarie M. C. van Rossum

**Affiliations:** 1 Department of Paediatric Infectious Diseases and Immunology, Erasmus MC–Sophia, Rotterdam, The Netherlands; 2 Laboratory of Paediatrics, Erasmus MC–Sophia, Rotterdam, The Netherlands; 3 Department of General Paediatrics, Erasmus MC–Sophia, Rotterdam, The Netherlands; 4 Department of Virology, Erasmus MC, Rotterdam, The Netherlands; 5 Department of Biostatistics, Erasmus MC, Rotterdam, The Netherlands; 6 Department of Plastic Surgery, Erasmus MC, Rotterdam, The Netherlands; 7 Department of Anaesthesiology, Erasmus MC, Rotterdam, The Netherlands; 8 General Practitioners Cooperative, Rotterdam, The Netherlands; 9 Department of General Practice, University of Groningen, University Medical Center Groningen, Groningen, The Netherlands; 10 Department of Medical Microbiology and Infectious Diseases, Erasmus MC, Rotterdam, The Netherlands; 11 Microbiology R&D, bioMérieux, La Balme-les-Grottes, France; Emory University, United States of America

## Abstract

**Background:**

*Mycoplasma pneumoniae* is thought to be a common cause of respiratory tract infections (RTIs) in children. The diagnosis of *M. pneumoniae* RTIs currently relies on serological methods and/or the detection of bacterial DNA in the upper respiratory tract (URT). It is conceivable, however, that these diagnostic methods also yield positive results if *M. pneumoniae* is carried asymptomatically in the URT. Positive results from these tests may therefore not always be indicative of a symptomatic infection. The existence of asymptomatic carriage of *M. pneumoniae* has not been established. We hypothesized that asymptomatic carriage in children exists and investigated whether colonization and symptomatic infection could be differentiated by current diagnostic methods.

**Methods and Findings:**

This study was conducted at the Erasmus MC–Sophia Children's Hospital and the after-hours General Practitioners Cooperative in Rotterdam, The Netherlands. Asymptomatic children (*n* = 405) and children with RTI symptoms (*n* = 321) aged 3 mo to 16 y were enrolled in a cross-sectional study from July 1, 2008, to November 30, 2011. Clinical data, pharyngeal and nasopharyngeal specimens, and serum samples were collected. The primary objective was to differentiate between colonization and symptomatic infection with *M. pneumoniae* by current diagnostic methods, especially real-time PCR. *M. pneumoniae* DNA was detected in 21.2% (95% CI 17.2%–25.2%) of the asymptomatic children and in 16.2% (95% CI 12.2%–20.2%) of the symptomatic children (*p* = 0.11). Neither serology nor quantitative PCR nor culture differentiated asymptomatic carriage from infection. A total of 202 children were tested for the presence of other bacterial and viral pathogens. Two or more pathogens were found in 56% (63/112) of the asymptomatic children and in 55.5% (50/90) of the symptomatic children. Finally, longitudinal sampling showed persistence of *M. pneumoniae* in the URT for up to 4 mo. Fifteen of the 21 asymptomatic children with *M. pneumoniae* and 19 of the 22 symptomatic children with *M. pneumoniae* in this longitudinal follow-up tested negative after 1 mo.

**Conclusions:**

Although our study has limitations, such as a single study site and limited sample size, our data indicate that the presence of *M. pneumoniae* in the URT is common in asymptomatic children. The current diagnostic tests for *M. pneumoniae* are unable to differentiate between asymptomatic carriage and symptomatic infection.

*Please see later in the article for the Editors' Summary*

## Abstract 2

In order to determine the possible asymptomatic carriage of *Mycoplasma pneumoniae* in the upper respiratory tracts of children, Emiel Spuesens and colleagues investigate the prevalence of *M. pneumoniae* in symptomatic and asymptomatic children at a hospital in The Netherlands.

*Please see later in the article for the Editors' Summary*

## Editors' Summary

**Background:**

Pneumonia (a form of acute respiratory infection) is the single largest cause of death in children worldwide, killing an estimated 1.2 million children aged five and under every year, particularly in South Asia and sub-Saharan Africa. In these settings, bacterial infections with *Streptococcus pneumoniae* and *Haemophilus influenzae* are the most common causes of bacterial pneumonia. However, in high-income settings, bacterial infection with *Mycoplasma pneumoniae* is a major cause of upper and lower respiratory tract infections in children: over one-third of childhood cases of community-acquired pneumonia that require admission to a hospital are caused by *M. pneumoniae*. Currently, diagnosis of *M. pneumoniae* infections relies on the detection of antibodies against *M. pneumoniae* in the blood or detection of bacterial DNA in samples from the upper respiratory tract through polymerase chain reaction (PCR) tests.

**Why Was This Study Done?:**

Other bacteria, such as *Streptococcus pneumoniae*, are commonly present in children without causing infection, a situation known as asymptomatic carriage. However, to date, it is unknown whether *M. pneumoniae* is also commonly carried in the upper respiratory tract of children without causing symptoms or leading to infection. The possibility of asymptomatic carriage of *M. pneumoniae* could have major implications for the interpretation of the results of diagnostic tests and also for clinical management. So in this study conducted in The Netherlands, the researchers investigated whether asymptomatic carriage of *M. pneumoniae* exists and also whether symptomatic infection could be differentiated from asymptomatic carriage by current diagnostic methods.

**What Did the Researchers Do and Find?:**

Between 2008 and 2011, the researchers recruited children aged between three months and 16 years attending a hospital in Rotterdam for an elective surgical procedure (asymptomatic group) or admitted with a respiratory tract infection (symptomatic group). All children had blood tests and respiratory samples (nasopharyngeal swab) taken on admission and were tested for other pathogens. The researchers invited children who tested positive for *M. pneumoniae* by PCR to attend for further follow-up and tested them monthly for the presence of *M. pneumoniae* DNA in the upper respiratory tract until the test was negative on two occasions. Using these methods, the researchers recruited 726 children over the study period—405 in the asymptomatic group and 321 in the symptomatic group. The researchers found that the prevalence of *M. pneumoniae* did not differ between the asymptomatic group and the symptomatic group, with prevalences of 21.2% and 16.2%, respectively (the prevalence of *M. pneumoniae* also did not differ significantly between those with lower versus upper respiratory infection). There were also no differences in prevalence in the asymptomatic and symptomatic groups when diagnosed using blood tests. The researchers found a high rate of multiple, coexisting bacterial and viral pathogens in both asymptomatic and symptomatic children: two or more pathogens were found in 56% (63/112) of the asymptomatic children and in 55.5% (50/90) of the symptomatic children. Furthermore, season and the year of enrollment affected the prevalence of *M. pneumoniae* in the asymptomatic group, ranging from 3% during the spring of 2009 to 58% during the summer of 2010. Finally, of the 21 children from the asymptomatic group who participated in the follow-up study, 15 (71%) tested negative within one month, and in the symptomatic group, 19 of 22 children (86%) tested negative after the first visit.

**What Do These Findings Mean?:**

These findings show that *M. pneumoniae* is carried at high rates in the upper respiratory tracts of healthy children, and that this asymptomatic carriage cannot be differentiated from symptomatic respiratory tract infection by diagnostic tests (serology or PCR). As the prevalence of *M. pneumoniae* varied between year and season, carriage of *M. pneumoniae* may follow a cyclic epidemic pattern. This study is from a single study site in one city in The Netherlands, with a relatively small number of children, and so these findings may not be generalizable to other populations. However, as this study suggests that current diagnostic tests do not discriminate between carriage and infection, clinicians may need to reconsider the clinical significance of a positive test result. Future studies are needed to address this diagnostic challenge and also to investigate possible factors that may affect the progression of asymptomatic carriage of *M. pneumoniae* to symptomatic infection.

**Additional Information:**

Please access these websites via the online version of this summary at http://dx.doi.org/10.1371/journal.pmed.1001444.

- MicrobeWiki has more information on *M. pneumoniae*
- Lab Tests Online explains current tests for *M. pneumoniae*

## Introduction

*Mycoplasma pneumoniae* is considered a major cause of upper and lower respiratory tract infections (RTIs) and respiratory tract disease (RTD) in humans, and particularly in children. Over one-third of the childhood cases of community-acquired pneumonia that require hospitalization are thought to be caused by *M. pneumoniae* [1],[2]. The current diagnosis of *M. pneumoniae* infections relies on the detection of either serum antibodies against *M. pneumoniae* or bacterial DNA in samples of the upper respiratory tract (URT), as recommended in the guidelines published by the British Thoracic Society and the Infectious Diseases Society of America [1],[2].

PCR-based methods are increasingly used in daily clinical practice, as well as in clinical studies, for the detection of *M. pneumoniae* because they provide fast and sensitive results in the acute phase of an infection [3]–[5]. However, RTD caused by other common bacterial pathogens (such as *Streptococcus pneumoniae*) cannot yet be diagnosed by PCR because these pathogens are asymptomatically carried in the human population at high rates. Likewise, if *M. pneumoniae* is commonly carried asymptomatically in the URT of children, the detection of this bacterial species may not indicate a symptomatic infection. This would have major implications for the interpretation of the results of current diagnostic methods for *M. pneumoniae* RTIs and their use in clinical management. Clinical management of *M. pneumoniae* RTI in children mainly consists of administration of macrolides, because *M. pneumoniae* is not susceptible to penicillins. In an increasing number of countries resistant strains of *M. pneumoniae* are rapidly emerging, and these are associated with prolonged disease. In Asia, up to 90% of *M. pneumoniae* derived from clinical samples is currently macrolide-resistant [6]. The frequent use of macrolides in children probably contributes significantly to the selection of macrolide-resistant strains [7]. Decreasing the use of macrolides by improving diagnostic methods, or their interpretation, might therefore help to prevent macrolide resistance.

In contrast to numerous published studies on carriage of *S. pneumoniae* in children, studies that specifically address asymptomatic carriage of *M. pneumoniae* have hitherto not been performed. To our knowledge, this is the first study in which the current state-of-the-art diagnostic method for *M. pneumoniae* RTI, i.e., PCR, is evaluated using a symptomatic and an asymptomatic group of children during a 3-y period of sampling. To our knowledge, this is also the first study to investigate the presence of *M. pneumoniae* in both symptomatic and asymptomatic children in a longitudinal fashion, including collecting data on the occurrence of symptomatic infection during carriage. Although previous studies have reported the presence of *M. pneumoniae* in seemingly healthy individuals, these studies all suffered from drawbacks related to the study design (such as the lack of an appropriate control group and/or the lack of a follow-up study) or to limitations of the diagnostic assays that were employed [8]–[15]. As a consequence, clear conclusions concerning the existence and dynamics of carriage of *M. pneumoniae* could thus far not be drawn. We hypothesized that asymptomatic carriage in children exists and investigated whether colonization and symptomatic infection could be differentiated by current diagnostic methods. Secondary objectives of the present study were to assess the possible association between acute symptomatic *M. pneumoniae* infection and children's age, and to determine the influence of *M. pneumoniae* genotype as well as viral and bacterial co-infections on the severity of *M. pneumoniae* RTIs.

## Methods

### Ethics Statement

This observational study was approved by the Medical Ethics Review Board of the Erasmus MC (NL20418.078.08) and was conducted at the Erasmus MC–Sophia Children's Hospital and the after-hours General Practitioners Cooperative in Rotterdam, The Netherlands. Written informed consent was obtained from all parents and from children above the age of 12 y.

### Study Design and Population

Study participants, aged 3 mo to 16 y, were enrolled between July 1, 2008, and November 30, 2011, in two groups. The first group, which will be referred to as “the asymptomatic group,” was enrolled during admission for a planned elective surgical procedure, unrelated to RTD, at the short-stay department of the hospital. Exclusion criteria were a current RTI (based on questionnaires and physical examination by the attending anesthesiologist), the use of antibiotics in the past 2 d (7 d for azithromycin), and severe concomitant disease (e.g., chronic lung disease, cardiovascular disease, neoplasia, liver disease, kidney disease, metabolic disease, or psychomotor impairment). Baseline characteristics (Table 1) and information about RTIs in the previous 2 mo were recorded using a standardized questionnaire. Two study-team members collected respiratory and blood samples just prior to the start of the surgical procedure, while the child was under general anesthesia. Three to four weeks later, a questionnaire was completed by phone on development of RTIs in the weeks after enrollment.

**Table 1:** Baseline characteristics of the 726 study participants.

Characteristic	Asymptomatic Group (*n* = 405)	Symptomatic Group		
		Total (*n* = 321)	Emergency Department (*n* = 131)	General Practitioners Cooperative (*n* = 190)
Age	5.17 (4.76)	2.65 (3.48)	2.18 (3.38)	2.97 (3.50)
Female	137 (34.1)	155 (48.0)	58 (44.3)	95 (50.0)
Immunizations	383 (96.0)	307 (95.3)	124 (95.4)	183 (96.3)
Parental Smoking	145 (36.4)	132 (41.1)	42 (31.8)	90 (47.6)
Family size ≥5	163 (39.6)	74 (23.2)	33 (25.2)	41 (21.6)
Day-care attendance	121 (30.5)	160 (50.5)	75 (58.1)	86 (45.7)
Prior RTI	129 (31.5)	NA	NA	NA
Lower RTI (including pneumonia)	NA	64 (20.4)	38 (29.5)	26 (14.1)
Pneumonia	NA	35 (10.9)	27 (20.6)	8 (4.2)
Hospitalization	NA	41 (13.5)	32 (25.2)	9 (5.1)

Data are *n* (percent), except for age, which is given as mean (standard deviation). “Immunizations” refers to being immunized per the national immunization program in The Netherlands.

NA, not applicable.

In the second group, i.e., “the symptomatic group,” children diagnosed with RTI were enrolled at either the emergency department of the hospital or the after-hours General Practitioners Cooperative. Exclusion criteria were the same as for the asymptomatic group, except for having a current RTI. A standardized questionnaire was used to record baseline characteristics, clinical symptoms, and diagnosis at the discretion of the attending physician. Respiratory specimens and a capillary blood sample were collected. Three to four weeks later, a second capillary blood sample was collected and information was recorded about the duration of the RTI, treatment, and hospitalization.

Children who tested positive for *M. pneumoniae* by PCR were invited to participate in a longitudinal follow-up study, from August 1, 2010, to November 30, 2011. After informed consent was obtained, each child was tested monthly for the presence of *M. pneumoniae* in the URT until the test was negative on two consecutive occasions.

### Study Procedures

From each child, a pharyngeal swab (BBL CultureSwab EZ, BD) was taken by gently stroking between the palatine arches superior to the tonsils. The swab was stored in phosphate-buffered saline. Subsequently, two nasopharyngeal specimens were taken. First, a flexible swab (Copan) was inserted into one nostril, guided to the posterior nasopharyngeal wall, removed, and stored in Amies transport medium. Second, 1 ml of normal saline was instilled into each nostril and then suctioned by a flexible catheter applied to a container. The catheter was rinsed with 2 ml of normal saline. Specimens were kept at 4°C until further preparation within hours of collection.

To prevent contamination of samples, we took the following precautions. First, the two members of the study team who performed all procedures tested *M. pneumoniae* PCR-negative throughout the study period. Second, samples were prepared and tested in different subunits of the laboratory. In a “nucleic acid–free” laboratory, the samples were divided in aliquots used for culture, nucleic acid isolation, and storage. In other laboratory subunits, samples were either cultured or used for nucleic acid isolation. Each step in the PCR procedure, i.e., the preparation of real-time PCR premixes, the addition of purified nucleic acids to these premixes, and the actual PCR reactions, were carried out in different laboratories. Finally, each PCR run contained positive and negative controls.

Detection of *M. pneumoniae* in the URT was performed by real-time PCR and culture on both the pharyngeal swabs and the nasopharyngeal washings. DNA isolation was performed on 200 µl of the original samples using the QIAamp DNA Mini Kit (Qiagen). A quantitative real-time (TaqMan) PCR assay was used to detect and quantify *M. pneumoniae* genomic DNA, as previously described [16]. Adequate negative control samples were included in each PCR run. Culture was performed using 100 µl of the original sample [17]. Molecular (sub)typing of *M. pneumoniae* was performed on *M. pneumoniae*–positive samples, using a pyrosequencing-based assay [16]. Rest material was stored at −80°C until further use.

Serum was stored at −80°C. Detection of anti–*M. pneumoniae*–specific antibodies was performed using Serion ELISA classic *M. pneumoniae* kits (Clindia Benelux).

The nasopharyngeal swabs were used for the detection of *S. pneumoniae*, *Staphylococcus aureus*, *Haemophilus influenzae*, and *Moraxella catarrhalis*, following standard microbiological procedures [18].

The batch-wise detection of viral nucleic acids was performed after the enrollment was closed. As all samples were stored at −80°C, selected samples were thawed. For the detection of viral nucleic acids, 60 µl of the original sample from a pharyngeal swab was diluted 10-fold with Dulbecco's Modified Eagle Medium to a total of 600 µl. Internally controlled nucleic acid extraction (input volume 200 µl, output volume 100 µl), subsequent (multiplex) real-time PCR, and its quality control were performed as described before [19]. A cycle threshold below 40 was considered a positive result. Viruses were determined in all *M. pneumoniae* PCR-positive children and in *M. pneumoniae* PCR-negative children matched with respect to month and year of sampling as well as age. There was missing data for 30 *M. pneumoniae*–positive participants, and these participants could therefore not be included in this analysis.

### Outcome Variables and Statistical Analyses

All data were analyzed using software package SPSS version 16.0.1. The protocol-defined objectives were as follows (Text S1): (1) to determine the distribution of genomic copy loads in both the asymptomatic group and the symptomatic group to differentiate carriage from infection and (2) to assess the age distribution of the presence of *M. pneumoniae* in both symptomatic children and asymptomatic children, and to determine the influence of *M. pneumoniae* genotype as well as viral and bacterial co-infections on the severity of *M. pneumoniae* RTIs.

The prevalence of a positive test result for *M. pneumoniae* was calculated in both groups and compared using the χ^2^ test. Groups were compared for the distribution of bacterial loads (genomic copy number) using the Mann-Whitney U test. *p*-Values below 0.05 were considered significant. Within the symptomatic group, a child was defined as having a *M. pneumoniae* infection when at least one of the samples from the child was found to be *M. pneumoniae*–positive by serology, culture, or PCR. Within the asymptomatic group, *M. pneumoniae* carriage was defined by a *M. pneumoniae*–positive result obtained by either culture or PCR.

The children in the symptomatic group with a *M. pneumoniae* infection were divided into two groups carrying the two *M. pneumoniae* subtypes (i.e., subtype 1 and subtype 2), and into two groups according to the presence of co-infections. Diagnosis (upper or lower RTI) and hospitalization were used as proxies for severity of disease. Groups were compared for these variables using the χ^2^ test.

To identify possible factors that could determine the presence of *M. pneumoniae*, subgroup analyses were done for variables that could reasonably have an effect on *M. pneumoniae* prevalence including age, gender, season and year of enrollment, family size, active or passive smoking, and day-care attendance. Additional variables in the symptomatic group were diagnosis and hospitalization. Additional variables in the asymptomatic group were RTI prior to enrollment and RTI just following enrollment. These variables were entered in multiple logistic regression analysis regardless of their bivariate association. Because these were all exploratory analyses, we did not adjust for multiple comparisons and *p*<0.05 was considered significant. To test for collinearity we calculated condition indices for the multivariate analyses. We found a condition index of 9.9 for the asymptomatic group and a condition index of 12.1 for the symptomatic group. Because both values are below the generally accepted warning signal of 15, and there were no strong correlations between the covariates, we believe that multicollinearity was not a major problem in this study.

To allow the inclusion of a sufficiently high number of *M. pneumoniae*–infected children below the age of 5 y, we used a precalculated sample size of 400 children ≤5 y and 100 children >5 y in the symptomatic group based on an estimated prevalence of *M. pneumoniae* of 10%. In the asymptomatic group we took the same number. After enrollment of a total of 412 children in the asymptomatic group, it was apparent that the study question on the existence of carriage could be addressed. This was due to a higher prevalence of *M. pneumoniae* than anticipated in the asymptomatic group and in the symptomatic group. Because additional enrollments would not have a significant influence on the outcomes of the study, it was deemed unethical to subject additional children to the study, and enrollment was subsequently discontinued.

## Results

**Figure 1:**
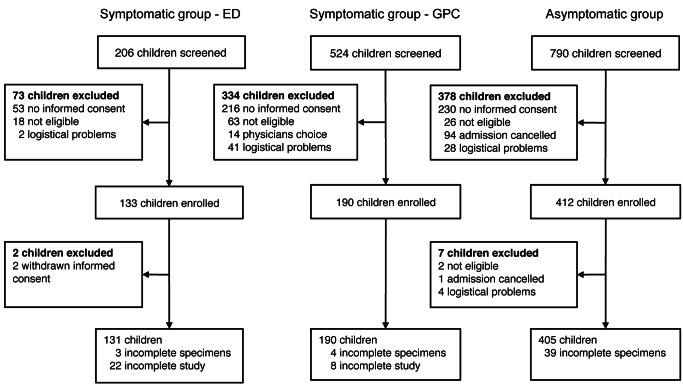
Enrollment flow diagram. ED, emergency department; GPC, General Practitioners Cooperative.

**Figure 2:**
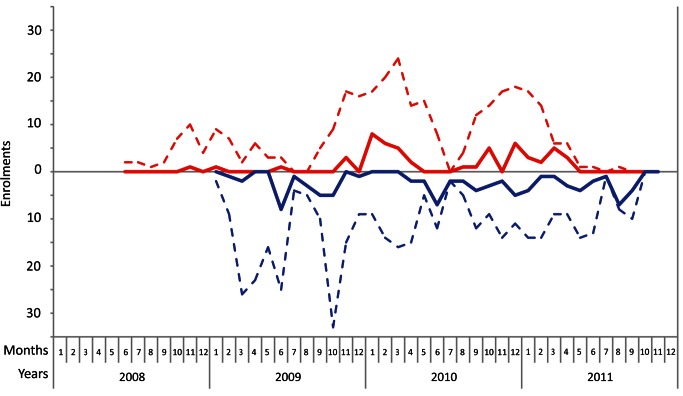
Monthly enrollments during the course of the study. The enrollments for the symptomatic group are represented above by a red dotted line. The enrollments for the asymptomatic group are represented below by a blue dotted line. The solid lines represent the absolute number of *M. pneumoniae*–positive participants. Enrollment for the asymptomatic group started in January 2009.

A total of 726 children, aged 3 mo to 16 y, were enrolled in this study from July 1, 2008, until November 30, 2011 (Figures 1 and 2). In the asymptomatic group, 405 children were enrolled. Enrollment for this group started in January 2009. In the symptomatic group, 321 children were enrolled. With respect to age, sex, and time of enrollment of study participants, there were no differences between the group of children for whom consent was given (enrolled in the study) and the group of children for whom consent was not given (Table S1).

We found that the prevalence of *M. pneumoniae* by real-time PCR did not differ significantly (*p* = 0.11) between the asymptomatic group (21.2%, 95% CI 17.2%–25.2%, *n* = 85) and the symptomatic group (16.2%, 95% CI 12.2%–20.2%, *n* = 51). A significant difference between the groups was also not found by culture: four (1.0%, 95% CI 0.03%–1.97%) of the asymptomatic children and five (1.6%, 95% CI 0.23%–2.97%) of the symptomatic children were *M. pneumoniae*–positive (*p* = 0.52). In the symptomatic group, the prevalence of *M. pneumoniae* by real-time PCR did not differ significantly (*p* = 0.85) between the children with a lower RTI (15.6%, 95% CI 11.6%–19.6%, *n* = 10) and the children with an upper RTI (15.9%, 95% CI 11.9%–19.9%, *n* = 41).

In the asymptomatic group, multiple logistic regression analysis showed that season and year of enrollment were significantly related to prevalence of *M. pneumoniae* (Tables 2 and S2). In the symptomatic group, we found the presence of *M. pneumoniae* to be positively associated with enrollment in 2010 and 2011 (Table S3). As shown in Table 3, none of the variables were independently related to the prevalence of *M. pneumoniae*. The presence of *M. pneumoniae* was not significantly associated with age or asthma-like symptoms (Tables 2 and 3). The two subtypes of *M. pneumoniae* were equally distributed between the two groups (Table S4).

**Table 2:** Results from the multiple logistic regression analysis for a positive *M. pneumoniae* PCR result in the asymptomatic group.

Variable	Odds Ratio (95% CI)	*p*-Value
**Age (≥5 y)**	0.96 (0.47–1.96)	0.91
**Gender (female)**	1.44 (0.81–2.56)	0.22
**Season**		<0.001 (overall)
Spring versus winter	0.81 (0.33–1.97)	0.64
Summer versus winter	7.43 (3.09–17.85)	<0.001
Autumn versus winter	2.90 (1.26–6.70)	0.01
**Year (2009 versus 2010 and 2011)**	3.31 (1.75–6.27)	<0.001
**Family size (≥5 family members)**	1.55 (0.77–3.13)	0.22
**Smoking (active or passive)**	0.70 (0.38–1.26)	0.23
**Presence or history of wheezing**	2.30 (0.69–7.61)	0.17
**Day-care attendance**	0.82 (0.38–1.77)	0.62
**RTI prior to enrollment**	0.95 (0.50–1.79)	0.86
**RTI post-enrollment**	0.59 (0.29–1.20)	0.15

The variable “immunizations” was not entered in the regression analysis because the vast majority of the children were immunized (>95%).

**Table 3:** Results from the multiple logistic regression analysis for a positive *M. pneumoniae* PCR result in the symptomatic group.

Variable	Odds Ratio (95% CI)	*p*-Value
**Age (≥5 y)**	1.56 (0.60–4.02)	0.36
**Gender (female)**	0.93 (0.46–1.87)	0.84
**Season**		0.87 (overall)
Spring versus winter	0.80 (0.35–1.81)	0.59
Summer versus winter	0.54 (0.11–2.67)	0.45
Autumn versus winter	0.85 (0.39–2.16)	0.74
**Year (2009 versus 2010 and 2011)**	5.80 (1.94–17.35)	0.002
**Family size (≥5 family members)**	1.63 (0.74–3.61)	0.23
**Smoking (active or passive)**	0.61 (0.30–1.23)	0.17
**Presence or history of wheezing**	1.96 (0.93–4.13)	0.08
**Day-care attendance**	0.84 (0.37–1.89)	0.67
**Diagnosis (lower RTI)**	1.05 (0.46–2.42)	0.91
**Hospitalization**	1.54 (0.52–4.60)	0.44

The variable “immunizations” was not entered in the regression analysis because the vast majority of the children were immunized (>95%). Symptoms and signs were not entered in the regression analysis because these are represented in the variable “diagnosis.”

**Figure 3:**
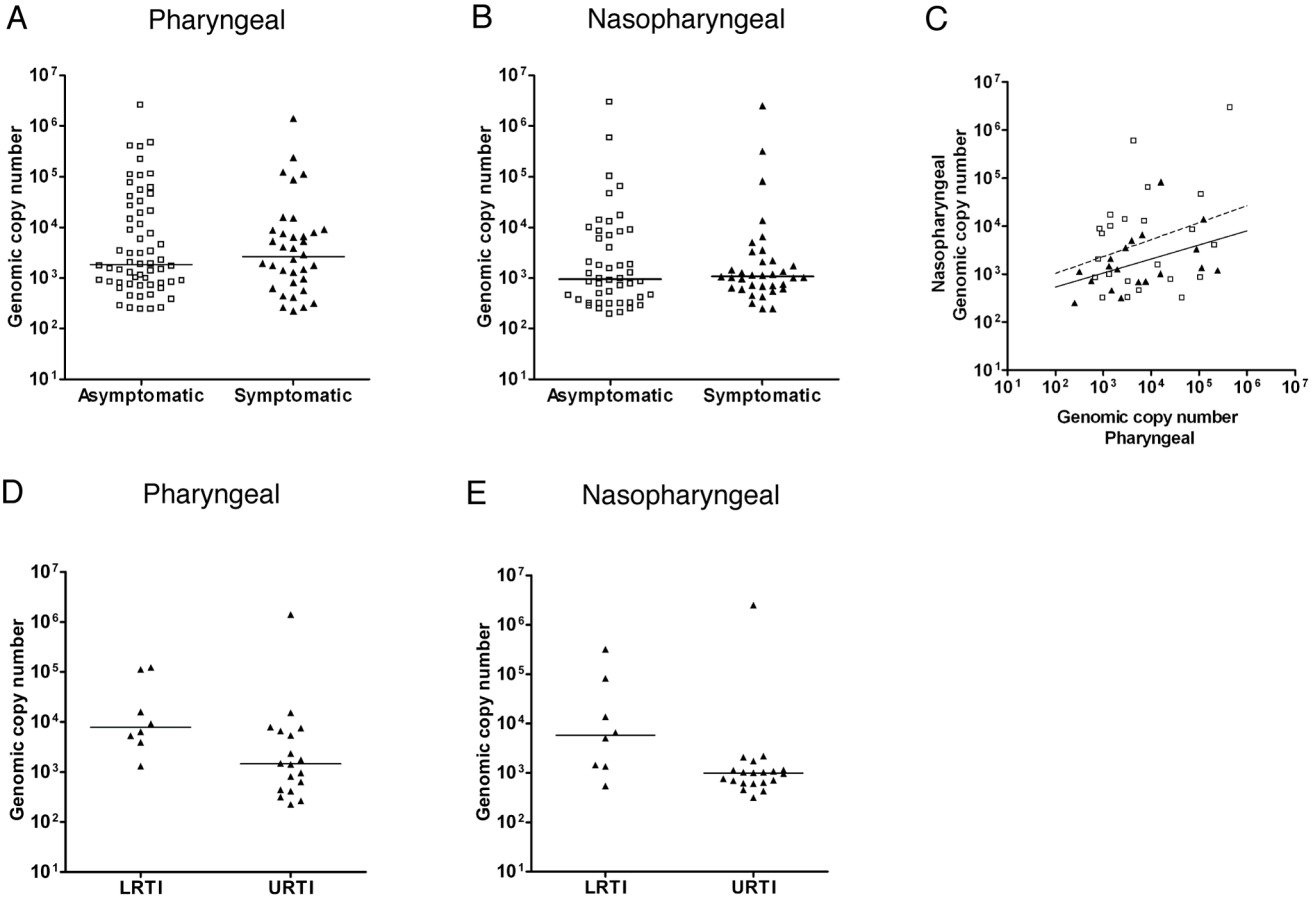
*M. pneumoniae* DNA loads. (A) Pharyngeal bacterial loads (genomic copy number per milliliter on the *y*-axis) of *M. pneumoniae* PCR-positive participants in the asymptomatic group (open squares) and the symptomatic group (filled triangles). (B) Nasopharyngeal bacterial loads (genomic copy number per milliliter on the *y*-axis) of *M. pneumoniae* PCR-positive participants in the asymptomatic group and the symptomatic group. The bacterial load distribution was compared using the Mann-Whitney U test. (C) Comparison of the bacterial loads in pharyngeal samples and nasopharyngeal samples for the participants who tested positive for *M. pneumoniae* in both. Correlation was calculated using the Spearman rank test. (D and E) Distribution of bacterial loads for upper RTIs (URTI) and lower RTIs (LRTI) in the pharyngeal and nasopharyngeal samples. The line in each graph represents the median.

A similar distribution of *M. pneumoniae* DNA loads was observed among the asymptomatic and symptomatic children (Figure 3A and 3B). We did not find a significant correlation between bacterial load in nasopharyngeal and pharyngeal samples (Figure 3C). The distribution of bacterial loads was different among children with a lower RTI and children with an upper RTI (Figure 3D and 3E). However, both very high and very low bacterial loads were detected in both subgroups. The study was not powered to perform a statistical subgroup analysis for this variable.

**Figure 4:**
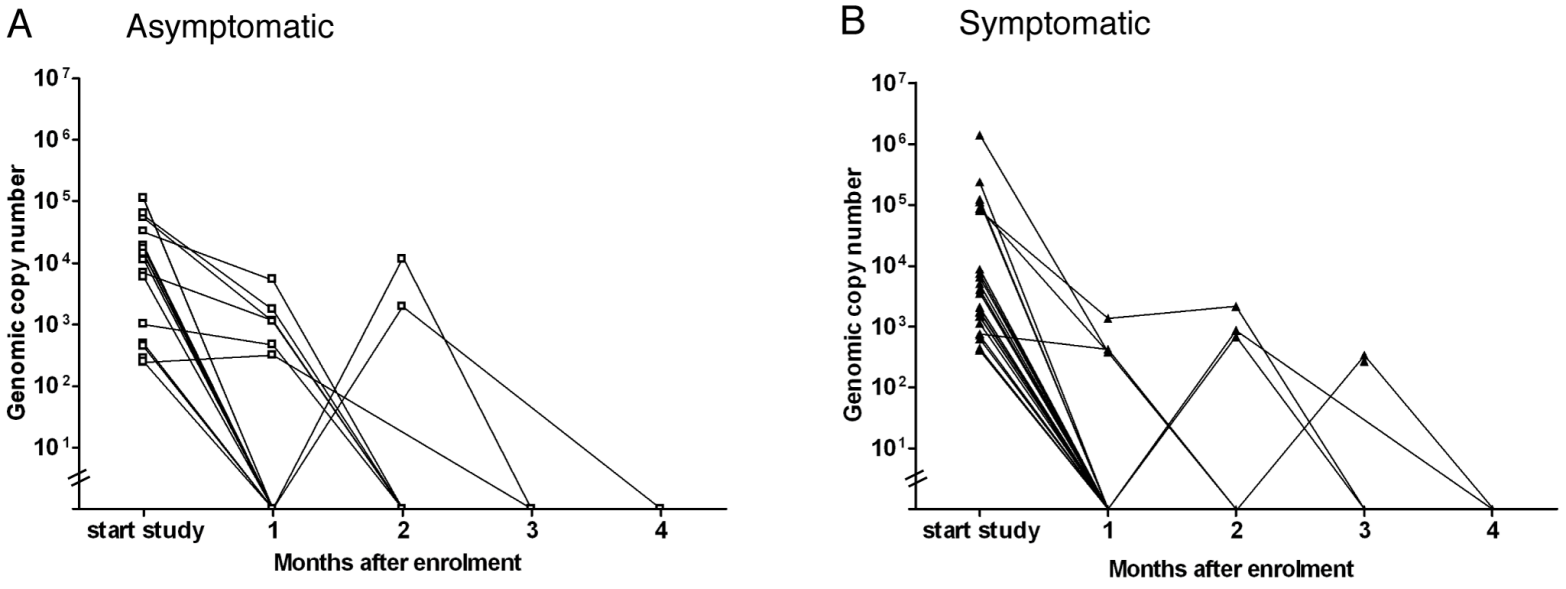
*M. pneumoniae* DNA loads in the longitudinal study. This figure shows the bacterial DNA loads in the study participants of the asymptomatic group (A) (open squares) and the symptomatic group (B) (filled triangles) during the follow-up study. Each point represents one visit of one participant and is connected by a line to the point representing the next visit. On the *y*-axis the bacterial DNA load (genomic copy number per milliliter) is shown. On the *x*-axis the consecutive visits are represented.

To investigate how long *M. pneumoniae* can persist in the respiratory tract of children, a longitudinal follow-up study was performed among children who tested positive for *M. pneumoniae* by PCR. In this study, 43 (68%) of the 63 children who were eligible for inclusion participated in the follow-up study. Of these 43 children, 21 children originated from the asymptomatic group, and 22 from the symptomatic group. The most common reason given for declining to participate in the follow-up study was distance from home to the study site (10/20; 50%). Fifteen of the 21 (71%) asymptomatic children and 19 of the 22 (86%) symptomatic children in this longitudinal follow-up study tested negative after 1 mo. Six of the asymptomatic children also tested positive at 2 mo, and two children also tested positive at 3 mo. Three of the symptomatic children tested positive at 2 mo, and none tested positive at 3 mo (Figure 4). To confirm their negative status, children were tested an additional time after becoming negative.

**Figure 5:**
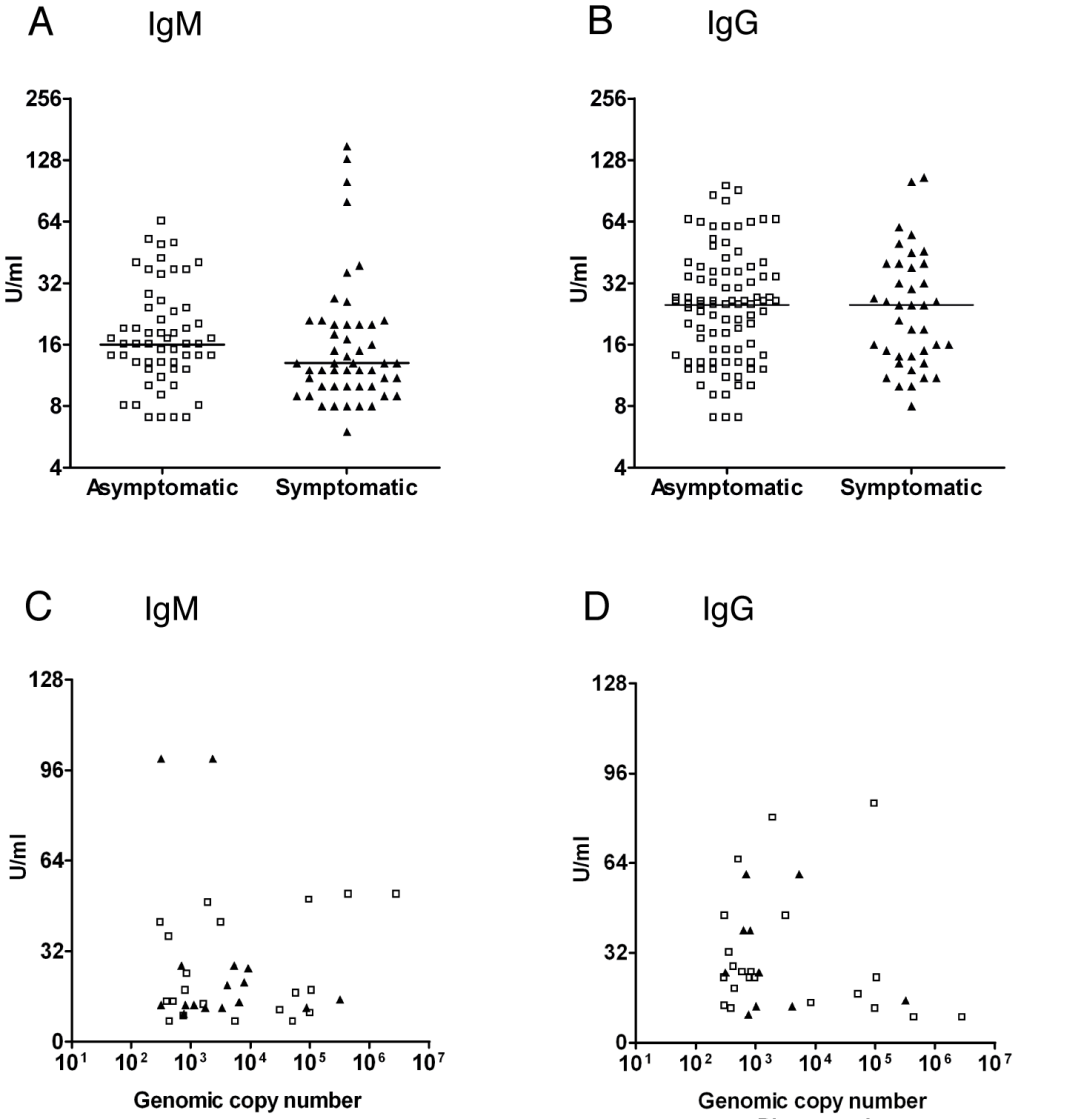
Anti–*M. pneumoniae* serum antibody levels. (A and B) Serum IgM (A) and IgG (B) antibody levels (in units/milliliter) are compared between the asymptomatic group and the symptomatic group (using the Mann-Whitney U test). (C and D) IgM (C) and IgG (D) antibody levels are plotted against the bacterial DNA load (genomic copy number per milliliter) in all samples. Open squares indicate asymptomatic participants. Filled triangles indicate symptomatic participants. The horizontal lines in A and B represent the median.

We found that the prevalence of a positive ELISA for anti–*M. pneumoniae* IgM antibodies was not significantly different in the asymptomatic group (12.6%, 95% CI 9.4%–15.8%, *n* = 43) and the symptomatic group (9.2%, 95% CI 6.0%–12.4%, *n* = 26) (*p* = 0.23). The prevalence of a positive ELISA for anti–*M. pneumoniae* IgG antibodies differed significantly (*p*<0.001) between the asymptomatic group (25.1%, 95% CI 20.9%–29.3%, *n* = 85) and the symptomatic group (14.2%, 95% CI 10.4%–18.0%, *n* = 40). However, when adjusted for age, there was no significant difference in the prevalence of anti–*M. pneumoniae* antibodies between the two groups. The prevalence of a positive ELISA for anti–*M. pneumoniae* IgM and IgG was low among children below the age of 5 y (IgM, 7.3%, 95% CI 3.5%–11.1%; IgG, 8.3%, 95% CI 4.3%–12.3%) in the asymptomatic group versus in the symptomatic group (IgM, 6.7%, 95% CI 0.0%–13.4%; IgG, 5.0%, 95% CI 0.0%–5.9%), and much higher among children above the age of 5 y (IgM, 20.4%, 95% CI 14.1%–26.7%, and IgG, 50.7%, 95% CI 42.8%–58.5%, in the asymptomatic group; IgM, 11.9%, 95% CI 7.7%–16.1%, and IgG, 47.6%, 95% CI 41.1%–54.1%, in the symptomatic group). The median levels of IgM and IgG antibodies did not differ significantly between the two groups (Figures 5A and 5B). The prevalence of a positive ELISA for anti–*M. pneumoniae* IgA antibodies was very low in both groups (2.0%, 95% CI 0.6%–3.4%, versus 0.4%, 95% CI 0.0%–1.1%), and none of the children below the age of 5 y tested positive for anti–*M. pneumoniae* IgA. The serological data did not correspond significantly with the PCR results (Figure 5C and 5D; Table 4). Interestingly, a higher percentage of asymptomatic PCR-positive children tested positive for IgG compared to symptomatic PCR-positive children (Table 4; 32% versus 15%). However, this difference was not found to be statistically significant when adjusted for age.

**Table 4:** Agreement between PCR and serology.

Test Result	Asymptomatic Group			Symptomatic Group		
	PCR		Kappa	PCR		Kappa
	Positive	Negative		Positive	Negative	
**Serology IgM**						
Positive	11	32		7	13	
Negative	55	242	0.06	40	215	0.12
**Serology IgA**						
Positive	4	3		0	1	
Negative	62	271	0.08	46	224	−0.01
**Serology IgG**						
Positive	16	68		6	25	
Negative	50	203	−0.01	41	202	0.02
**Immunoglobulin class switch**						
Positive	NA	NA		7	19	
Negative	NA	NA		31	176	0.10

NA, not applicable.

Because an immunoglobulin class switch from IgM to IgG is generally accepted as evidence of a recent *M. pneumoniae* infection, a second serum sample was collected from 233 (72.6%) of the symptomatic children. From this group, 26 (11.2%) children developed an immunoglobulin class switch. The agreement between an immunoglobulin class switch and a positive PCR result was poor (Kappa = 0.10) (Table 4). Besides age, we did not find any determinants of an immunoglobulin class switch or a single positive test for IgM, IgG, or IgA.

**Figure 6:**
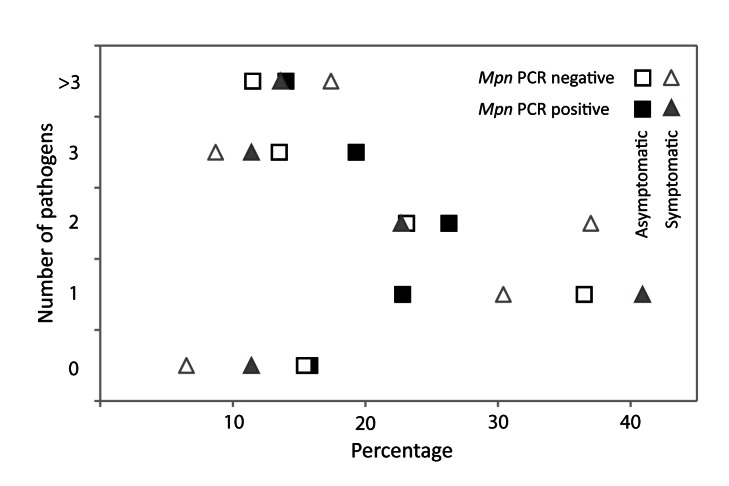
Number of detected viral and bacterial pathogens. The dot plot shows the percentages of participants with 0, 1, 2, 3, or >3 pathogens present in the URT. On the *x*-axis the percentages are shown, on the *y*-axis the number of pathogens is shown. The filled and open triangles show respectively *M. pneumoniae* (*Mpn*) PCR-positive symptomatic children (*n* = 44) and *M. pneumoniae* PCR-negative symptomatic children (*n* = 46). The filled and open squares show respectively *M. pneumoniae* PCR-positive asymptomatic children (*n* = 57) and *M. pneumoniae* PCR-negative asymptomatic children (*n* = 52).

Bacterial and viral pathogens do coexist in the respiratory tract, and co-infection may influence severity of disease. Therefore, we determined the presence of four other bacterial respiratory pathogens in all children and the presence of 15 viral respiratory pathogens in a subsample of the children (*n* = 202). Two or more pathogens were found in 56% (63/112) of the asymptomatic children and in 55.5% (50/90) of the symptomatic children. The prevalence of the four bacterial pathogens is shown in Table 5. As reported previously, the prevalence of these bacterial species was largely age-dependent [20]. We found a significantly higher prevalence of *S. aureus* in the asymptomatic group than in the symptomatic group. However, when adjusted for age this difference in prevalence was not significant. An association between the presence of any of these four bacterial species and *M. pneumoniae* was not detected. Almost all of the viruses screened for were detected in both groups (Table 6). We did not perform a statistical analysis to detect differences in the distribution of cycle threshold values since our study was not powered for this purpose. Rhinovirus, bocavirus, and parainfluenzavirus 4 were detected more frequently in asymptomatic children than in symptomatic children. In contrast, influenzaviruses A and B, human metapneumovirus, and respiratory syncytial virus were predominantly detected in symptomatic children. The majority of the children (from both groups) tested positive for more than one pathogen (Figure 6). Group-specific combinations of pathogens could not be identified. In addition, none of the viruses were associated with the presence of *M. pneumoniae*. Because of the limited number of *M. pneumoniae*–positive children without bacterial and/or viral co-infection, it was not possible to analyze the influence of bacterial and viral co-infection on disease severity.

**Table 5:** Bacterial results in 714 study participants.

Bacterium	Asymptomatic Participants (*n* = 393)	Symptomatic Participants (*n* = 321)	*p*Value
*Str. pneumoniae*	109 (27.7)	87 (27.1)	0.92
*S. aureus*	84 (21.4)	32 (10.0)	<0.001
*M. catarrhalis*	71 (18.1)	74 (23.1)	0.12
*H. influenzae*	57 (14.5)	51 (15.9)	0.68

Data are *n* (percent). The *p*-values compare the difference in prevalence between the two participant groups indicated by χ^2^ test.

**Table 6:** Virology results in 202 study participants.

Virus	Asymptomatic Participants (*n* = 112)		Symptomatic Participants (*n* = 90)		*p*-Value
	Ct Value, Median (IQR)	*n* (Percent)	Ct Value, Median (IQR)	*n* (Percent)	
Influenza A virus	20.1	1 (0.9)	23.0 (18.0–33.8)	6 (6.7)	0.03
Influenza B virus	Undetectable	0 (0.0)	26.0	1 (1.1)	0.26
Human metapneumovirus	36.7–38.7	2 (1.8)	24.6 (23.0–30.5)	7 (7.8)	0.10
Respiratory syncytial virus A	27.9	1 (0.9)	22.0 (20.0–29.6)	11 (12.2)	0.001
Respiratory syncytial virus B	35.4	1 (0.9)	22.0 (17.3–29.5)	9 (10.0)	0.003
Parainfluenzavirus 1	36.3 (22.0–37.6)	7 (6.3)	25.3 (20.0–26.5)	3 (3.3)	0.34
Parainfluenzavirus 2	35.8 (34.6–35.9)	3 (2.7)	31.1	1 (1.1)	0.43
Parainfluenzavirus 3	20.7. 35.8	2 (1.8)	30.6 (25.1–39.6)	3 (3.3)	0.48
Parainfluenzavirus 4	36.5 (31.9–37.4)	10 (8.9)	38.7	1 (1.1)	0.02
Rhinovirus	26.5 (23.1–31.1)	35 (31.2)	24.7 (22.3–29.4)	17 (18.9)	0.04
Coronavirus 229E	Undetectable	0 (0.0)	17.6	1 (1.1)	0.26
Coronavirus OC43	35.6 (28.7–37.3)	5 (4.5)	29.0 (24.1–32.9)	5 (5.6)	0.72
Coronavirus NL63	37.5 (34.8–38.6)	6 (5.4)	27.5 (27.0–38.0)	7 (7.8)	0.49
Bocavirus	30.9 (26.5–34.0)	16 (14.3)	32.0 (28.8–34.0)	3 (3.3)	0.008
Adenovirus	31.9 (27.9–33.8)	17 (15.2)	27.0 (26.2–30.5)	9 (10.0)	0.28

The *p*-values compare the difference in prevalence between the two participant groups indicated by χ^2^ test.

Ct value, cycle threshold value; IQR, interquartile range.

## Discussion

### Statement of Principal Findings

To our knowledge, our study demonstrates for the first time that *M. pneumoniae* is carried at high rates in the URT of healthy children, and that this asymptomatic carriage cannot be differentiated from symptomatic RTI by serology or quantitative PCR. Of 405 healthy children, 21% tested positive for *M. pneumoniae* in the URT by PCR. As a result of this high prevalence, the inclusion of children was terminated at an earlier time point than anticipated at the start of the study. For now we can conclude that *M. pneumoniae* carriage is detectable and its prevalence is higher than expected, but the actual prevalence of carriage is unreliable. Prevalence varied between year and season of sampling from 3% during the spring of 2009 to 58% during the summer of 2010. These data suggest that carriage follows a cyclic epidemic pattern. It is tempting to speculate that this fluctuation in prevalence is related to the known cyclic epidemic pattern of *M. pneumoniae* infections that occurs at intervals of 3–7 y, in addition to a background endemic pattern [4],[21]. Longitudinal sampling of *M. pneumoniae*–positive asymptomatic children indicated that *M. pneumoniae* can be present in the URT without causing disease, followed by clearance within several weeks.

While previous studies have demonstrated the presence of *M. pneumoniae* in seemingly healthy individuals, none of these studies could draw clear conclusions concerning the actual existence of carriage of *M. pneumoniae* [8]–[15]. Instead, some studies explained the presence of *M. pneumoniae* in an asymptomatic individual as a consequence of a recent infection with this bacterium [14],[15]. Other studies were inconclusive because of lack of a specific study design or because of the use of relatively insensitive diagnostic tools such as culture [9]–[12].

### Strengths and Weaknesses

Although we have demonstrated the existence of asymptomatic carriage of *M. pneumoniae* in children, there are several limitations to our study. These include the single study site in one city in the Netherlands, and a limited sample size. Although the sample size was adequate to address our research questions, it is not large enough to unravel the dynamics of colonization by *M. pneumoniae*. Furthermore, we performed exploratory analyses that show that *M. pneumoniae* prevalence is determined by season and year of sampling, although these analyses did not form part of the original design of the study. Finally, we have performed a longitudinal follow-up study that shows the persistence of *M. pneumoniae* in a small number of children for up to 4 mo. Although this finding strongly suggests the existence of carriage, we enrolled only a small number of children in this part of the study. Future studies are needed to confirm our results and should aim at finding determinants of *M. pneumoniae* carriage. These studies should preferably have a multicenter design.

Furthermore, we aimed to assess the association between severity of disease and *M. pneumoniae* subtype and genomic copy load. Although we did not find this association in the symptomatic group, it should be noted that participants positive for *M. pneumoniae* subtype 2 were underrepresented overall (as shown in Table S4). This small number hampered the analysis of this secondary research question. It might also be argued that these results are negatively influenced by a general low severity of disease in our study population and the limited number of participants with lower RTI (20.9% of all symptomatic children). Indeed, none of the children in our study were admitted to an intensive care unit or required respiratory support by mechanical ventilation. Still, we did find a difference between asymptomatic and symptomatic children for well-established respiratory pathogens such as influenza A and respiratory syncytial virus. We think that this point underlines the appropriateness of our study population with regard to severity.

In agreement with other recent studies on *M. pneumoniae* infections, a poor correlation was found between data obtained by PCR and serology [22],[23]. Given the excellent performance of the PCR assay in Quality Control for Molecular Diagnostics panels, and the validation of the commercial ELISA used, we are confident that the data obtained by both assays are reliable. It is possible that the positive serological results simply reflect one or more previous encounters with *M. pneumoniae* and are not necessarily related to a current RTI or carriage of *M. pneumoniae* as determined by PCR. However, we did not collect convalescent serum samples in the asymptomatic group; therefore, we can only speculate on the prevalence of immunoglobulin class switch or the levels of convalescent antibodies in this group.

### Meaning of the Study

As the pathogenicity of *M. pneumoniae* was well-documented in studies with volunteers in the 1950s [24], one can speculate that asymptomatic carriage of *M. pneumoniae* may in some cases lead to symptomatic infection, as is well known to occur for other pathogens [25]. Obviously, the finding of asymptomatic carriage of *M. pneumoniae* has major implications for the interpretation of the diagnosis of *M. pneumoniae* infections and for clinical management, as well as the interpretation of studies of the etiology of RTD in children. We searched Medline with the terms “*Mycoplasma pneumoniae*,” “respiratory tract infection,” “asymptomatic carriage,” and “diagnosis” and found that many studies and some reviews have addressed the performance and clinical value of different diagnostic methods for *M. pneumoniae*. It is striking, however, that these methods are used interchangeably, and almost every positive result is regarded as indicative of a symptomatic *M. pneumoniae* infection. In addition, the occasional presence of *M. pneumoniae* in respiratory secretions of healthy individuals has often been explained either as the first sign of a developing symptomatic infection or as bacterial persistence following symptomatic infection [3],[11],[26]–[28]. Current guidelines on community-acquired pneumonia recommend testing for *M. pneumoniae* in patients with a high pre-test probability [1],[2]. As we have shown, the available procedures for diagnosis of *M. pneumoniae* RTIs in children do not discriminate between carriage of *M. pneumoniae* in the respiratory tract and symptomatic *M. pneumoniae* infection. Therefore, clinicians may need to readdress the clinical significance of a positive test result.

Our data indicate that the etiology of RTI in children is complex. The mere presence of one or more putative pathogens in the URT does not seem to be the sole determining factor in the development of a symptomatic RTI. The host immune response, the timing of colonization, the presence of other pathogens, and the initial bacterial or viral load may collectively determine whether carriage proceeds to infection or not. Future studies will therefore have to focus on how an RTI can be accurately defined and whether or not it requires treatment.

### Conclusion and Future Studies

The results of this study suggest that *M. pneumoniae* behaves similarly to many other bacterial species in the respiratory tract. Future studies at different sites and in different populations are required to confirm our findings. This finding is important as it implies that the daily clinical practice of diagnosing *M. pneumoniae* RTI is inadequate. Specifically, it does not seem appropriate to use the detection of *M. pneumoniae* in the URT by PCR as a method to diagnose symptomatic RTIs caused by this bacterium. Thus, a diagnosis of *M. pneumoniae*–induced RTD cannot be based exclusively on serology or the detection of *M. pneumoniae* DNA in the URT, and caution should be taken in the interpretation of diagnostic tests for *M. pneumoniae*. Future studies should address this diagnostic challenge and aim at finding diagnostic tools that can differentiate carriage from infection, as well as identifying factors that may determine progression from asymptomatic carriage of *M. pneumoniae* to symptomatic infection.

## Supporting Information

Table S1 Comparison between the children for whom consent was given (enrolled in the study) and the children for whom consent was not given.
(DOC)

Table S2 Bivariate analysis in the asymptomatic group. This table shows the prevalence of *M. pneumoniae* as determined by PCR for the variables age, gender, immunizations, season of enrollment, year of enrollment, family size, smoking, presence or history of wheezing, day-care attendance, RTI prior to enrollment, and RTI in the month after enrollment.
(DOC)

Table S3 Bivariate analysis in the symptomatic group. This table shows the prevalence of *M. pneumoniae* as determined by PCR for the variables age, gender, immunizations, season of enrollment, year of enrollment, family size, smoking, presence or history of wheezing, day-care attendance, symptoms present at the time of enrollment, diagnosis (presence of a lower RTI), and hospitalization.
(DOC)

Table S4 The distribution of the different *M. pneumoniae* genotypes (i.e., subtype 1 and 2) in the asymptomatic and symptomatic groups.
(DOC)

Text S1 Study protocol.
(DOC)

## Abbreviations

RTD: respiratory tract disease
RTI: respiratory tract infection
URT: upper respiratory tract
