# Supplementary material for: Carriage of Mycoplasma pneumoniae in the Upper Respiratory Tract of Symptomatic and Asymptomatic Children: An Observational Study
Source: PLoS Med. 2013 May 14;10(5):e1001444. doi: 10.1371/journal.pmed.1001444 (PMC3653782; doi:10.1371/journal.pmed.1001444)
Supplement: Table S1 — Comparison between the children for whom consent was given (enrolled in the study) and the children for whom consent was not given. (DOC) [file pmed.1001444.s001.doc]

**Table S1. Prevalence of age, gender and season at enrolment for the group for which consent was given versus the group for which consent was not given**

| **Category** | **Subcategory** | **Asymptomatic % (n/N)** | | | **Symptomatic % (n/N)** | | |
| --- | --- | --- | --- | --- | --- | --- | --- |
|  |  | **Consent** | **No consent ^a,b^** | **p-value** | **Consent** | **No consent ^c,d^** | **p-value** |
| **Age** | **< 5** | 50.1 (230/459) | 49.9 (229/459) | 0.15 | 46.4 (260/560) | 53.6 (300/560) | 0.14 |
|  | **≥ 5** | 55.3 (182/329) | 44.7 (147/329) |  | 40.1 (61/152) | 59.9 (91/152) |  |
| **Gender** | **F** | 52.8 (272/515) | 47.2 (243/515) | 0.31 | 47.8 (153/320) | 52.2 (167/320) | 0.88 |
|  | **M** | 51.3 (140/273) | 48.7 (133/273) |  | 48.0 (168/350) | 52.0 (182/350) |  |
| **Season** | **Winter** | 46.4 (84/181) | 53.6 (97/181) | 0.32 | 42.5 (122/287) | 57.5 (165/287) | 0.10 |
|  | **Spring** | 55.3 (135/244) | 44.7 (109/244) |  | 38.0 (84/221) | 62.0 (137/221) |  |
|  | **Summer** | 51.6 (79/153) | 48.4 (74/153) |  | 41.5 (22/53) | 58.5 (31/53) |  |
|  | **Autumn** | 53.8 (114/212) | 46.2 (98/212) |  | 55.0 (93/169) | 45.0 (76/169) |  |

a. Of 2 children in the asymptomatic no consent group, age was not recorded. b. Of 2 children in the asymptomatic no consent group, gender was not recorded. c. Of 18 children in the symptmatic no consent group, age was not recorded. d. Of 60 children in the symptomatic no consent group, gender was not recorded.
