# Supplementary material for: Carriage of Mycoplasma pneumoniae in the Upper Respiratory Tract of Symptomatic and Asymptomatic Children: An Observational Study
Source: PLoS Med. 2013 May 14;10(5):e1001444. doi: 10.1371/journal.pmed.1001444 (PMC3653782; doi:10.1371/journal.pmed.1001444)
Supplement: Table S2 — Bivariate analysis in the asymptomatic group. This table shows the prevalence of M. pneumoniae as determined by PCR for the variables age, gender, immunizations, season of enrollment, year of enrollment, family size, smoking, presence or history of wheezing, day-care attendance, RTI prior to enrollment, and RTI in the month after enrollment. (DOC) [file pmed.1001444.s002.doc]

**Table S2. Prevalences of *M. pneumoniae*** in the asymptomatic group

| **Category** | **Subcategory** | ***M. pneumoniae* PCR positive % (n/N)** |
| --- | --- | --- |
| **Age** | **< 5** | 21.4 (48/224) |
|  | **≥ 5** | 20.8 (37/172) |
| **Gender** | **F** | 19.2 (51/265) |
|  | **M** | 24.8 (34/137) |
| **Immunizations** | **Complete** | 21.6 (83/384) |
|  | **Incomplete or none** | 0.0 (0/5) |
| **Season** | **Winter** | 14.6 (12/82) |
|  | **Spring** | 10.5 (14/133) |
|  | **Summer** | 43.8 (32/73) |
|  | **Autumn** | 23.9 (27/113) |
| **Year of enrolment** | **2009** | 14.7 (26/177) |
|  | **2010** | 23.4 (29/124) |
|  | **2011** | 30.0 (30/100) |
| **Family size** | **< 5** | 18.8 (61/325) |
|  | **≥ 5** | 30.7 (23/75) |
| **Smoking** | **No** | 22.8 (56/246) |
|  | **Active or passive** | 18.5 (28/151) |
| **Presence or history of wheezing** | **None** | 20.8 (78/375) |
|  | **Yes** | 30.4 (7/23) |
| **Daycare attendance** | **No** | 21.7 (59/272) |
|  | **Yes** | 20.2 (25/124) |
| **RTI prior enrolment** | **No** | 22.3 (61/274) |
|  | **Yes** | 18.4 (23/125) |
| **RTI post enrolment** | **No** | 26.4 (68/258) |
|  | **Yes** | 18.6 (16/86) |

RTI respiratory tract infections
