# Supplementary material for: Carriage of Mycoplasma pneumoniae in the Upper Respiratory Tract of Symptomatic and Asymptomatic Children: An Observational Study
Source: PLoS Med. 2013 May 14;10(5):e1001444. doi: 10.1371/journal.pmed.1001444 (PMC3653782; doi:10.1371/journal.pmed.1001444)
Supplement: Table S3 — Bivariate analysis in the symptomatic group. This table shows the prevalence of M. pneumoniae as determined by PCR for the variables age, gender, immunizations, season of enrollment, year of enrollment, family size, smoking, presence or history of wheezing, day-care attendance, symptoms present at the time of enrollment, diagnosis (presence of a lower RTI), and hospitalization. (DOC) [file pmed.1001444.s003.doc]

**Table S3. Prevalences of *M. pneumoniae* in the symptomatic group**

| **Category** | **Subcategory** | ***M. pneumoniae* PCR positive % (n/N)** |
| --- | --- | --- |
| **Age** | **< 5** | 14.6 (37/253) |
|  | **≥ 5** | 23.0 (14/61) |
| **Gender** | **F** | 17.2 (26/151 |
|  | **M** | 15.3 (25/163) |
| **Immunizations** | **Complete** | 16.7 (50/300) |
|  | **Incomplete or none** | 0.0 (0/3) |
| **Season** | **Winter** | 19.7 (24/122) |
|  | **Spring** | 19.5 (15/77) |
|  | **Summer** | 9.1 (2/22) |
|  | **Autumn** | 10.8 (10/93) |
| **Year of enrolment** | **2008** | 3.6 (1/28) |
|  | **2009** | 6.5 (5/77) |
|  | **2010** | 20.9 (34/163) |
|  | **2011** | 23.9 (11/46) |
| **Family size** | **< 5** | 15.0 (36/240) |
|  | **≥ 5** | 20.8 (15/72) |
| **Smoking** | **No** | 16.2 (29/179) |
|  | **Active or passive** | 15.5 (20/129) |
| **Presence or history of wheezing** | **None** | 15.5 (32/207) |
|  | **Yes** | 19.0 (19/100) |
| **Daycare attendance** | **No** | 20.0 (30/150) |
|  | **Yes** | 12.2 (19/156) |
| **Rhinorrhea** | **No** | 23.2 (26/112) |
|  | **Yes** | 12.4 (25/202) |
| **Sore throat** | **No** | 16.5 (43/261) |
|  | **Yes** | 17.0 (8/47) |
| **Earache** | **No** | 14.5 (37/256) |
|  | **Yes** | 24.6 (14/57) |
| **Headache** | **No** | 16.3 (49/300) |
|  | **Yes** | 14.3 (2/14) |
| **Myalgia** | **No** | 16.0 (50/312) |
|  | **Yes** | 50.0 (1/2) |
| **Cough** | **No** | 16.9 (14/83) |
|  | **Yes** | 16.0 (37/231) |
| **Fever** | **No** | 15.8 (15/95) |
|  | **Yes** | 16.4 (36/219) |
| **Lower respiratory tract infection** | **No** | 15.6 (10/64) |
|  | **Yes** | 16.6 (40/241) |
| **Hospitalisation** | **No** | 17.1 (7/41) |
|  | **Yes** | 16.5 (41/248) |
