# Supplementary material for: Carriage of Mycoplasma pneumoniae in the Upper Respiratory Tract of Symptomatic and Asymptomatic Children: An Observational Study
Source: PLoS Med. 2013 May 14;10(5):e1001444. doi: 10.1371/journal.pmed.1001444 (PMC3653782; doi:10.1371/journal.pmed.1001444)
Supplement: Table S4 — The distribution of the different M. pneumoniae genotypes (i.e., subtype 1 and 2) in the asymptomatic and symptomatic groups. (DOC) [file pmed.1001444.s004.doc]

**Table S4. *M. pneumoniae* subtypes in asymptomatic and symptomatic children**

|  |  | **Asymptomatic** | **Symptomatic** |
| --- | --- | --- | --- |
| ***M. pneumoniae* subtype** | **1** | 31 (75.6) | 25 (83.3) |
|  | **2** | 10 (24.4) | 5 (16.7) |
