# Supplementary material for: Carriage of Mycoplasma pneumoniae in the Upper Respiratory Tract of Symptomatic and Asymptomatic Children: An Observational Study
Source: PLoS Med. 2013 May 14;10(5):e1001444. doi: 10.1371/journal.pmed.1001444 (PMC3653782; doi:10.1371/journal.pmed.1001444)

**Colonization and infection**

**of the respiratory tract by**

***Mycoplasma pneumoniae* in children**

**PROTOCOL TITLE**

**Colonization and infection of the respiratory tract by *Mycoplasma pneumoniae* in children**

| **Protocol ID** | **MymIC-studie** |
| --- | --- |
| **Short title** | ***Mycoplasma Pneumoniae*: Infection and Colonization** |
| **Version** | **5** |
| **Date** | **03-06-2010** |
| **Coordinating investigator/project leader** | **Dr. A.M.C. van Rossum, pediatrician**  **Erasmus MC Sophia Rotterdam**  Department of Pediatrics Infectious Diseases/Immunology  **Dr. Molewaterplein 60**  **3015 GJ Rotterdam**  **The Netherlands**  **Tel. +31 10 704 0 704**  **Fax. +31 10 703 68 11**  **E-mail: a.vanrossum@erasmusmc.nl** |
| Principal investigator(s) (hoofdonderzoeker/uitvoerder) | **Department of Pediatrics, Erasmus MC Sophia Children’s Hospital**  **Dr. Molewaterplein 60**  **3015 GJ Rotterdam**  **Principal Investigator: Dr. A.M.C. van Rossum, pediatrician**  **Investigator: Drs. E.B.M. Spuesens, research physician**  **Laboratory of Pediatrics**  **Postbus 2040**  **3000 CA Rotterdam**  **Principal investigator: Dr. C. Vink** |
| **Sponsor (verrichter/opdrachtgever)** | **None** |
| **Independent physician(s)** | **Drs. B.J. Sibbles – van Genderen, pediatrician**  **Email: b.sibbles@erasmusmc.nl** |
| **Laboratory sites** | **Erasmus MC Sophia**  Department of Pediatric Infectious Diseases/Immunology  **Dr. Molewaterplein 60**  **3015 GJ Rotterdam**  **Principal Investigator: Dr. A.M.C. van Rossum, pediatrician**  **Investigator: Drs. E.B.M. Spuesens, research physician**  **Laboratory of Pediatrics**  **Postbus 2040**  **3000 CA Rotterdam**  **Principal Investigator: Dr. C. Vink, Medical Microbiologist** |
| **Pharmacy** | **Not Applicable** |
|  |  |

**PROTOCOL SIGNATURE SHEET**

| **Name** | **Signature** | **Date** |
| --- | --- | --- |
| **Sponsor or legal representative:**  **For non-commercial research,**  **Head of Department:**  **Prof. Dr. A.J. van der Heijden** |  |  |
| **Coordinating Investigator/Project leader/Principal Investigator:**  **Dr. A.M.C. van Rossum** |  |  |
|  |  |  |

**TABLE OF CONTENTS**

1 INTRODUCTION AND RATIONALE [10](#__RefHeading___Toc137193414)

2 OBJECTIVES…………………………………………………………………………………… 11

3 STUDY DESIGN [13](#__RefHeading___Toc137193416)

4 STUDY POPULATION [15](#__RefHeading___Toc137193418)

4.1 Population (base) [15](#__RefHeading___Toc137193419)

4.2 Inclusion criteria [15](#__RefHeading___Toc137193420)

4.3 Exclusion criteria [15](#__RefHeading___Toc137193421)

4.4 Sample size calculation [15](#__RefHeading___Toc137193422)

5 TREATMENT OF SUBJECTS [16](#__RefHeading___Toc137193423)

5.1 Investigational product/treatment [16](#__RefHeading___Toc137193424)

5.2 Escape medication [16](#__RefHeading___Toc137193425)

6 INVESTIGATIONAL MEDICINAL PRODUCT [17](#__RefHeading___Toc137193426)

6.1 Name and description of investigational medicinal product [17](#__RefHeading___Toc137193427)

6.2 Summary of findings from non-clinical studies [17](#__RefHeading___Toc137193428)

6.3 Summary of findings from clinical studies [17](#__RefHeading___Toc137193429)

6.4 Summary of known and potential risks and benefits [17](#__RefHeading___Toc137193430)

6.5 Description and justification of route of administration and dosage [17](#__RefHeading___Toc137193431)

6.6 Dosages, dosage modifications and method of administration [17](#__RefHeading___Toc137193432)

6.7 Preparation and labelling of Investigational Medicinal Product [17](#__RefHeading___Toc137193433)

6.8 Drug accountability [17](#__RefHeading___Toc137193434)

7 METHODS [18](#__RefHeading___Toc137193435)

7.1 Study parameters/endpoints [18](#__RefHeading___Toc137193436)

*Secondary study parameters/endpoints* [18](#__RefHeading___Toc137193437)

*Other study parameters* [18](#__RefHeading___Toc137193438)

7.2 Randomisation, blinding and treatment allocation [18](#__RefHeading___Toc137193439)

7.3 Study procedures [18](#__RefHeading___Toc137193440)

*Specific criteria for withdrawal* [18](#__RefHeading___Toc137193441)

Not applicable [18](#__RefHeading___Toc137193442)

7.4 Replacement of individual subjects after withdrawal [18](#__RefHeading___Toc137193443)

Not applicable [18](#__RefHeading___Toc137193444)

7.5 Follow-up of subjects withdrawn from treatment [19](#__RefHeading___Toc137193445)

Not applicable [19](#__RefHeading___Toc137193446)

7.6 Premature termination of the study [19](#__RefHeading___Toc137193447)

8 SAFETY REPORTING [20](#__RefHeading___Toc137193448)

8.1 Section 10 WMO event [20](#__RefHeading___Toc137193449)

8.2 Adverse and serious adverse events [20](#__RefHeading___Toc137193450)

8.2.1 Suspected unexpected serious adverse reactions (SUSAR) [20](#__RefHeading___Toc137193451)

8.2.2 Annual safety report [20](#__RefHeading___Toc137193452)

8.3 Follow-up of adverse events [20](#__RefHeading___Toc137193453)

8.4 Data Safety Monitoring Board (DSMB) [20](#__RefHeading___Toc137193454)

9 STATISTICAL ANALYSIS [21](#__RefHeading___Toc137193455)

10 ETHICAL CONSIDERATIONS [22](#__RefHeading___Toc137193456)

10.1 Regulation statement [22](#__RefHeading___Toc137193457)

10.2 Recruitment and consent [22](#__RefHeading___Toc137193458)

10.3 Objection by minors or incapacitated subjects [22](#__RefHeading___Toc137193459)

10.4 Benefits and risks assessment, group relatedness [22](#__RefHeading___Toc137193460)

10.5 Compensation for injury [22](#__RefHeading___Toc137193461)

10.6 Incentives [22](#__RefHeading___Toc137193462)

11 ADMINISTRATIVE ASPECTS AND PUBLICATION [23](#__RefHeading___Toc137193463)

11.1 Handling and storage of data and documents [23](#__RefHeading___Toc137193464)

11.2 Amendments [23](#__RefHeading___Toc137193465)

11.3 Annual progress report [23](#__RefHeading___Toc137193466)

11.4 End of study report [23](#__RefHeading___Toc137193467)

11.5 Public disclosure and publication policy [23](#__RefHeading___Toc137193468)

12 Ancillary studies [24](#__RefHeading___Toc137193469)

12.1 Ancillary study 1: Follow-up study to investigate carriage. [24](#__RefHeading___Toc137193470)

12.1.1 Rationale [24](#__RefHeading___Toc137193471)

12.1.2 Objective [24](#__RefHeading___Toc137193472)

12.1.3 Study design [24](#__RefHeading___Toc137193473)

12.1.4 Study population [25](#__RefHeading___Toc137193476)

12.1.5 Study Methods [26](#__RefHeading___Toc137193481)

12.1.6 Statistics [26](#__RefHeading___Toc137193482)

13 REFERENCES [27](#__RefHeading___Toc137193483)

14 APPENDIX [28](#__RefHeading___Toc137193484)

14.1 Flowchart FUP study [28](#__RefHeading___Toc137193485)

**LIST OF ABBREVIATIONS AND RELEVANT DEFINITIONS**

| **ABR** | **ABR form (General Assessment and Registration form) is the application form that is required for submission to the accredited Ethics Committee (ABR = Algemene Beoordeling en Registratie)** |
| --- | --- |
| **AE** | **Adverse Event** |
| **AR** | **Adverse Reaction** |
| **CA** | **Competent Authority** |
| **CCMO** | **Central Committee on Research Involving Human Subjects** |
| **CV** | **Curriculum Vitae** |
| **DSMB** | **Data Safety Monitoring Board** |
| **EU** | **European Union** |
| **EudraCT** | **European drug regulatory affairs Clinical Trials GCP Good Clinical Practice** |
| **IB** | **Investigator’s Brochure** |
| **IC** | **Informed Consent** |
| **IMP** | **Investigational Medicinal Product** |
| **IMPD** | **Investigational Medicinal Product Dossier** |
| **METC** | **Medical research ethics committee (MREC); in Dutch: medisch ethische toetsing commissie (METC)** |
| **(S)AE** | **Serious Adverse Event** |
| **SPC** | **Summary of Product Characteristics (in Dutch: officiële productinfomatie IB1-tekst)** |
| **Sponsor** | **The sponsor is the party that commissions the organisation or performance of the research, for example a pharmaceutical**  **company, academic hospital, scientific organisation or investigator. A party that provides funding for a study but does not commission it is not regarded as the sponsor, but referred to as a subsidising party.** |
| **SUSAR** | **Suspected Unexpected Serious Adverse Reaction** |
| **Wbp** | **Personal Data Protection Act (in Dutch: Wet Bescherming Persoonsgevens)** |
| **WMO** | **Medical Research Involving Human Subjects Act (Wet Medisch-wetenschappelijk Onderzoek met Mensen** |

**SUMMARY**

Rationale

150 million children/year suffer from pneumonia worldwide, and 20 million children have to be hospitalized for this reason. Up to 40% of community-acquired pneumonias are caused by Mycoplasma pneumoniae and as many as 34% of cases requiring hospitalization in children. Studies using the recently introduced PCR suggest that M. pneumoniae frequently causes respiratory tract infections (RTI), not only in older children as previously thought, but also in children younger than 5 years. This is important because M. pneumoniae is not sensitive to the first choice β-lactam antibiotics. Moreover, it is unknown whether the detection of M. pneumoniae by PCR also confirms this pathogen as the cause of the infection. A limited number of studies suggest the existence of an asymptomatic carrier state. Due to the shortcomings in diagnosis, knowledge on the role of different host- and bacterial factors on progression to infection is very limited.

Hypothesis

M. pneumoniae is capable of asymptomatic colonization, which can be differentiated from infection by quantitative PCR. M. pneumoniae causes RTI’s in children younger than 5 years as frequently as in older children. The genetic background of M. pneumoniae strains as well as viral co-infections influence progression from colonization to infection.

Objectives

1. To optimize the diagnosis of M. pneumoniae infections by discriminating between colonization and symptomatic infection using quantitative PCR.

2. To study the role of host factors (age, and bacterial and viral co-infection) on infection by M. pneumoniae.

3. To investigate the relationship between M. pneumoniae genotype and virulence.

Study design

Prospective design

Study population

Group A: children with a suspected RTI

Group B: healthy controls

Intervention

Not Applicable.

Main study parameters/endpoints

In this explorative study, M. pneumoniae will be quantified in children aged 0-16 years with mild to severe symptoms of respiratory tract infection, and in a control group consisting of children without respiratory tract infection. The quantity of M. pneumoniae (in copies/ml or copies/swab) will be related to clinical disease in order to differentiate asymptomatic carrier state from infection. To investigate the role of co-colonization or -infection, the presence of other bacteria or viruses in the respiratory tract will be analyzed as well. To study the age distribution in relation to M. pneumoniae infection, a precalculated number of patients of either < 5 or ≥ 5 years of age will be included. Genotyping of M. pneumoniae will be performed, and related to severity of disease and/or colonization. Prevalence of colonization and/or infection with M. pneumoniae will be calculated.

From all study participant the following samples will be obtained:

- Oropharyngeal swab
- Nasopharyngeal swab
- Nasopharyngeal lavage
- Serum

Obtaining these samples is considered common clinical practice. The risks and burden are negligible.

# INTRODUCTION AND RATIONALE

# Lower respiratory tract infections (LRTIs) are among the most common infections in children and adults. The World Health Organization estimates there are 150.7 million cases of pneumonia each year in children younger than 5 years, with as many as 20 million cases severe enough to require hospital admission. Respiratory tract infections are the number one killer in children in developing countries. (1) In children with lower respiratory tract infections, M. pneumoniae is the second most common cause of LRTI after Streptococcus pneumoniae. (2) M. pneumoniae is a human pathogen that causes a range of respiratory infections, such as tracheobronchitis, pharyngitis, and atypical pneumonia. Up to 40% of community-acquired pneumonias are caused by M. pneumoniae and as many as 34% of cases requiring hospitalization in children. (3, 4) Glomerulonephritis, carditis, rhabdomyolysis, Bell palsy, Guillain-Barre syndrome, and acute transverse myelitis are rare complications of M. pneumoniae infection. In addition, infection with M. pneumoniae is associated with both chronic stable asthma and acute exacerbations of asthma. (5) Infection with M. pneumoniae is therefore a major burden of disease. This study has three objectives.

1. A specific diagnosis of the cause of LRTI is important because β-lactam antibiotic treatment of M. pneumoniae infections is ineffective, due to the lack of a cell wall, whereas the use of antibiotics such as macrolides can markedly reduce the duration of the illnesses. (6) While the clinical diagnosis of LRTI is usually relatively straightforward, determining the etiological diagnosis can be much more difficult due to the limitations of conventional diagnostic tests, and because of difficulty in obtaining adequate sputum samples for culture in children. As culture of M. pneumoniae is slow and insensitive, the laboratory diagnosis has largely relied on serological testing. Although there have been recent improvements, the sensitivity and specificity of antibody detection is suboptimal. In addition, it often only provides a retrospective diagnosis of acute infection because a convalescent serum specimen is needed to show a fourfold increase in titre. Serologic testing is therefore not optimum for patient management, however this is still used as golden standard for M. pneumoniae detection. (7)

More recently, polymerase chain reaction (PCR) demonstrated the potential to produce rapid, sensitive and specific results, and is now considered the method of choice for direct pathogen detection. (8) However, there is justified concern when the PCR assay is used as the sole means of detection without culture, serology, or clinical data, because surveillance studies using culture and/or PCR indicate that a prolonged asymptomatic carrier state may occur in some persons. (9-12) Since it is not known whether there is a specific threshold quantity of M. pneumoniae in respiratory tract tissues that can differentiate asymptomatic carrier state from infection, a positive result by PCR may overestimate the clinical importance, and may result in unnecessary use of antibiotics. On the other hand, if quantitative PCR will demonstrate to be a reliable technique for diagnosing M. pneumoniae infection, M. pneumoniae will be diagnosed more often. This will result in more adequate treatment of RTI’s in children. It has been suggested that children colonized with M. pneumoniae might be an unrecognized reservoir for the spread of this pathogen. (12) The application of quantitative PCR assays will be crucial in optimizing diagnosing RTI by M. pneumoniae, and in gaining a better understanding of the carrier state associated with M. pneumoniae. Therefore, this is one of the main objectives of our study.

1. M. pneumoniae is thought to be a pathogen that causes pneumonia in children aged 5 years and older. Choice of treatment is based on this supposed age-distribution. Since M. pneumoniae is not susceptible to β-lactam antibiotics, a macrolide antibiotic is the first choice treatment in this age-group. In children younger than 5 years, β-lactam antibiotics are the first choice treatment, because M. pneumoniae is presumed to be a rare cause of RTI in this age-group. However, data on age distribution are conflicting. (13) Specialized diagnostic techniques showed that M pneumoniae might have a more important role in causing both upper and lower respiratory tract infections than previously thought; they seem to be frequent also in children aged less than 5 years. (4, 14) To address this issue, we will investigate the rate of infection among children belonging to different age groups.

In addition, we will study the role of bacterial and viral co-infections. Co-infections can account for up to 30-50% of the cases of M pneumoniae infection. (4, 15, 16) Infection with M. pneumoniae is frequently associated with infections with S. pneumoniae and respiratory viruses. This association might be due to damage to the respiratory epithelium by a virus facilitating infection with M. pneumoniae. However, virtually nothing is known regarding this issue. Children younger than 5 years frequently suffer from viral respiratory infections, which might support the data that M. pneumoniae infections in this age-group are more frequent than previously thought. It is also unknown whether viral co-infection leads to more severe clinical disease. This study aims to answer these questions.

1. A crucial step in the initiation of infection by M. pneumoniae is its attachment to the respiratory epithelium (cytadherence). This process is essential to pathogenesis since mutants that are unable to adhere are also avirulent.(17) Cytadherence is mediated by a specialized and complex attachment organelle. This organelle is localized at the tip of the bacterium and consists of a network of adhesins and accessory proteins. The major adhesion protein (cytadhesin) that is concentrated in the attachment organelle is the surface-exposed, 170-kDa P1 protein. Apart from its function in binding to the respiratory epithelium, the P1 protein is known to elicit a strong humoral immune response during infection. (18) In relation to its immunodominance, the P1 protein was hypothesized to undergo antigenic variation on the basis of the presence within its gene of sequences of which multiple variants exist throughout the M. pneumoniae genome. (19, 20) It is therefore possible that recombination between the P1 gene and these sequences elsewhere in the genome could generate significant sequence variation within the P1 gene, resulting in amino acid changes in the P1 protein at the bacterial surface. This proposed mechanism for antigenic variation of P1 may provide a means for M. pneumoniae to escape from host immune responses. (21) Our research group in the Laboratory of Pediatrics aims to design a definitive, universal system for typing of M. pneumoniae strains on the basis of the P1 sequences, and to understand antigenic variation of the P1 protein. Together, we will aim to elucidate the relationship between M. pneumoniae virulence and P1 antigenic variation using the collected samples, and clinical data.
2. **OBJECTIVES**

Our objectives are to:

1. Optimize the diagnosis of M. pneumoniae infections by differentiating between asymptomatic colonization and infection using quantitative, real-time PCR
2. Study host factors for infection with M. pneumoniae (age distribution of children suffering from M. pneumoniae infections, and the role of co-infection with common bacterial pathogens, and respiratory viruses)
3. Determine the influence of genotypic differences between M. pneumoniae strains on virulence.

# STUDY DESIGN

**Aim I: To optimize the diagnosis of M. pneumoniae infections using quantitative PCR**

We will initiate our investigations in a well-defined and easy accessible study population. Children visiting the Emergency Department of the Erasmus Medical Center/Sophia Children’s Hospital in Rotterdam because of suspected respiratory tract infection will be asked to participate in this study. Since this department has an open environment, many children present with symptoms of both upper and lower respiratory tract infections, submitted by a general practitioner as well as by themselves or their parents. In fall 2009 the recruitment of group A will be extended by pediatric patients attending the General Practitioner’s Service, in this protocol referred to as HAP (Huis Artsen Post).

Group B, consisting of healthy controls, will be recruited from children undergoing surgery at the Erasmus MC/ Sophia Children’s Hospital. Many children need to have surgical procedures for reasons that are not in any way related to the exclusion criteria of our study. These children, who have not suffered from an RTI over the previous two weeks and who have not used antibiotics in the preceding 48 hours, are considered eligible for inclusion. The main reason to address these children instead of approaching schools and day care centres is the fact that children undergoing a surgical procedure are drugged by anaesthetics anyway. This way the burden for healthy controls will be minimized.

*Clinical samples*

From each patient the following samples will be obtained

- Oropharyngeal swab
- Nasopharyngeal swab
- Nasopharyngeal lavage
- Serum (blood sample of 200 microliters)

In group A second serum samples will be taken 2-3 weeks after the first visit.

*Clinical data*

For each patient, patient characteristics, time of onset of illness, clinical signs and symptoms, use of antibiotics, laboratory results and radiological findings, treatment, complications, and hospitalization will be documented in a standardized questionnaire. Based on clinical data different diagnosis will be made. This will be divided in lower respiratory tract infections (such a pneumonia and bronchiolitis) and upper respiratory tract infections (such as pharyngitis). Severity of disease will be determined by days of required oxygen or mechanical ventilation.

*Detection of M. pneumoniae*

All swabs will be washed out in a buffer, which allows bacterial culturing as well as total nucleic acid (DNA and RNA) purification. Material will be frozen at -80°C and analyzed as a batch. Purified nucleic acids will be used in real-time PCR assays for the detection of M. pneumoniae.

M. pneumoniae will be detected and quantified using a quantitative, real-time (TaqMan®) PCR assay that has recently been developed in the Laboratory of Pediatrics by Dr. C. Vink.

RTI by M. pneumoniae will be defined by RTI symptoms in combination with serology and/or culture positive for M. pneumoniae and/or conventional PCR for M. pneumoniae.

Aim II: To study the role of host factors (age, and bacterial and viral co-infection) on infection by M. pneumoniae

*Approach*

See aim I

*Clinical samples*

See aim I

*Clinical data*

See aim I. Virulence will be defined by disease severity (determined by hospitalization and duration of hospitalization).

*Detection of M. pneumoniae and other bacteria and viruses:*

See aim I. The other bacteria that will be investigated are S. pneumoniae, Staphylococcus aureus, Haemophilus Influenzae and Moraxella catarrhalis. These bacteria will be detected using routine, conventional bacterial culturing. In collaboration with the department of virology (Prof. Dr. A.D.M.E. Osterhaus), the following respiratory viruses will be detected by real-time PCR: influenzaviruses, parainfluenzaviruses, rhinoviruses, respiratory syncitial virus (RSV), coronaviruses and human metapneumovirus (hMPV).

**Aim III: To investigate the role of M. pneumoniae genotype on virulence**

*Approach*

See aim I

*Clinical samples*

See aim I

*Clinical data*

See aim II.

*Detection of M. pneumoniae*

See aim I

*Genotyping of the M. pneumoniae P1 gene*:

To type all isolated M. pneumoniae strains, the complete P1 gene of each strain will be amplified by PCR and sequenced. For each strain, at least two independently generated PCR fragments will be sequenced in order to check for potential PCR artefacts. The obtained sequences will be compared to each other and to sequences deposited in the GenBank database (http://www.ncbi.nlm.nih.gov/entrez/query.fcgi?db=Nucleotide&itool=toolbar). Based on the different sequences of the P1 gene, an M. pneumoniae strain classification scheme will be determined and a typing method will be designed. Specific genotypes will be compared to patient data, in order to relate genotype to bacterial virulence.

# Collaboration: influenza research

Concurrently to our study an influenza trial is being conducted in the Erasmus MC-Sophia. Due to the amount of similar research goals and methods, both studies are aiming to obtain the same patient population and same samples. Close collaboration between the research teams enables patients to be less burdened by our studies: parents only need to read one patient information leaflet instead of two separate ones. Moreover, blood sampling is conjugated: blood sampling will be performed on one occasion and after sampling, the blood is split and divided between the researchers. The only difference when participating in both the influenza study and the mycoplasma study is the quantity of the sample. Instead of the 0,5 ml blood which is required for the mycoplama study; 3 to 5 ml blood is wanted for the influenza study. Parents and children need to give their explicit consent before entering both studies: it is stated in the consent form.

#

# STUDY POPULATION

## Population (base)

Age: 0-16 years

Group A: 500 children visiting the Emergency Department/HAP because of upper or lower respiratory tract infection, from which: 400 children <5 years and 100 children ≥ 5 years.

Group B: 500 healthy controls; recruited from children attending the outpatient clinic in preparation of their surgery. Again: 400 children <5 years and 100 children ≥ 5 years.

## Inclusion criteria

Group A

- Age: ≥3 months and ≤ 16 years
- Clinical signs and symptoms of community-acquired upper or lower respiratory tract infection (i.e. cough, rhinitis, sore throat, wheezing and fever).
- Written informed consent

Group B (control group)

- Age: ≥3 months and ≤ 16 years
- Written informed consent
- Healthy controls: absence for ≥1 week of clinical signs and symptoms of community-acquired upper or lower respiratory tract infection (i.e. cough, rhinitis, sore throat, wheezing and fever)

## Exclusion criteria

Group A

- Age: ≤ 3 months and ≥ 16 years
- use of antibiotics less than 48 hours preceding enrolment
- use of azithromycin within 1 week preceding enrolment.
- Severe concomitant disease (chronic lung disease, neoplasia, liver or kidney disease, immunodeficiency, cardiovascular disease, psychomotor impairment, nosocomial infection).
- No written informed consent

Group B (control group)

- Age: ≤ 3 months and ≥ 16 years
- Clinical signs and symptoms of community-acquired upper or lower respiratory tract infection (i.e. cough, rhinitis, sore throat, wheezing, and/or fever).
- use of antibiotics less than 48 hours preceding enrolment
- use of azithromycin within 1 week preceding enrolment.
- Severe concomitant disease (chronic lung disease, neoplasia, liver or kidney disease, immunodeficiency, cardiovascular disease, psychomotor impairment, nosocomial infection).
- No written informed consent

## Sample size calculation

For estimated group sizes in 2 years of 500 in group A and 500 in group B small differences in mean quantitative result of PCR will be demonstrable (α = 0.05, power ≥ 90%) if the effect-size (difference of mean/standard deviation) is 0.20 or larger, which can be considered as a small difference. Group A we aim to include pre-calculated numbers according to age (400 children, < 5 years, versus 100 children, > 5 years). Because of a high prevalence viral RTI below 5 years of age, it is necessary to include more children with RTI in this group, to find a reasonable number of M. pneumoniae infections. Assuming up to 10% M. pneumoniae infections below 5 years of age we will be able to detect a 12% difference using these numbers in these subgroups (< 5 years versus >5 years). For comparison we aim to include the same numbers in group B (healthy children).

# TREATMENT OF SUBJECTS

This entire section is not applicable

## Investigational product/treatment

Use of co-intervention

## Escape medication

# INVESTIGATIONAL MEDICINAL PRODUCT

This entire section is not applicable.

## Name and description of investigational medicinal product

## Summary of findings from non-clinical studies

## Summary of findings from clinical studies

## Summary of known and potential risks and benefits

## Description and justification of route of administration and dosage

## Dosages, dosage modifications and method of administration

## Preparation and labelling of Investigational Medicinal Product

## Drug accountability

# METHODS

## Study parameters/endpoints

*Main study parameter/endpoint*

- Differentiation between asymptomatic colonization and infection by M. pneumoniae by using quantitative real time PCR.
- Age distribution of M. pneumoniae.
- Relation of genotype, co-infections or co-colonization and virulence of M. pneumoniae.

### *Secondary study parameters/endpoints*

Not applicable

### *Other study parameters*

Not applicable

## Randomisation, blinding and treatment allocation

Not applicable.

## Study procedures

Visit 1:

From each patient the following samples will be obtained

- Oropharyngeal swab
- Nasopharyngeal swab
- Nasopharyngeal lavage
- Serum (blood sample of 200 microliters)

Patients from group A will have their samples taken during their visit at the Emergency Department/HAP. In those cases many of the samples are taken for diagnostic reasons anyway. Samples can usually be split for clinical (diagnostic) and research purposes. This will minimize the burden for the patients.

Patients from group B are at the day care ward for surgical treatment. The blood sample will be drawn from the vena puncture, which is conducted by the anaesthesiologist, in order to obtain iv-access for administration of anaesthetics drugs. The other samples are taken after the patient has had their anaesthetics.

Patients from both groups fill out a short questionnaire about their health (history, use of antibiotics, smoking, attendance of day care centre, etc)

Visit 2:

Group A will be asked to return for a follow-up visit, 3 tot 4 weeks after their initial visit. During this visit another blood sample will be taken and there will be another short questionnaire.

Group B will not have to return to hospital. They will be phoned by a member of the research team to check if there were any respiratory tract infections in our healthy control or his/her family since the day of surgery.

Withdrawal of individual subjects

Subjects can leave the study at any time for any reason if they wish to do so without any consequences. The investigator can decide to withdraw a subject from the study for urgent medical reasons.

### *Specific criteria for withdrawal*

## Not applicable

## Replacement of individual subjects after withdrawal

## Not applicable

## Follow-up of subjects withdrawn from treatment

## Not applicable

## Premature termination of the study

Not applicable

# SAFETY REPORTING

## Section 10 WMO event

In accordance to section 10, subsection 1, of the WMO, the investigator will inform the subjects and the reviewing accredited METC if anything occurs, on the basis of which it appears that the disadvantages of participation may be significantly greater than was foreseen in the research proposal. The study will be suspended pending further review by the accredited METC, except insofar as suspension would jeopardise the subjects’ health. The investigator will take care that all subjects are kept informed.

## Adverse and serious adverse events

Not applicable

### Suspected unexpected serious adverse reactions (SUSAR)

Not applicable

### Annual safety report

Not applicable

## Follow-up of adverse events

Not applicable

## Data Safety Monitoring Board (DSMB)

Not applicable

# STATISTICAL ANALYSIS

Research questions and analysis

Question 1 (objective 1): does asymptomatic carrier state with M. pneumoniae exist (see question 3, we expect to find > 10%, using quantitative PCR) (10), and if so, can asymptomatic carrier state be differentiated from infection using quantitative PCR?

Analysis: in children with respiratory tract infections caused by M. pneumoniae (as defined by positive standard PCR, positive serology, and or positive culture, group A), results from the quantitative PCR (results in copies/ml) will be compared with the results from the quantitative PCR in asymptomatic children (healthy asymptomatic children with positive results from either standard conventional PCR or culture, group B) using the Mann-Whitney U test. A PCR value, which can differentiate asymptomatic carrier state from infection (threshold), will be determined using Receiver Operating Characteristic (ROC) curves. This value should have a high sensitivity and acceptable specificity.

Question 2 (objective 1): what is the sensitivity and specificity of this quantitative PCR?

Analysis: sensitivity and specificity will be determined using Receiver Operating Characteristic (ROC) curves. In addition we will investigate the sensitivity and specificity according to whether the quantitative PCR results in a positive or negative result (lowest possible detecting range is 10 copies).

Question 3 (objective 1): what is the prevalence of asymptomatic carrier state?

Analysis: prevalence of asymptomatic carrier state (a positive result with quantitative PCR) will be calculated in group A (children with a positive quantitative PCR result and RTI not caused by M. pneumoniae) and B (healthy asymptomatic children with a positive PCR result). We expect to find >10% in group B (10).

Question 4 (objective 2): does M. pneumoniae infection occur more often in children older than 5 years?

Analysis: children in group A with a RTI caused by M. pneumoniae will be divided in two groups, according to age (< 5 years and ≥ 5 years) and compared to each other, with respect to the prevalence using Χ^2^-test.

Below five years of age there’s a high prevalence of viral infections. It is therefore necessary to use a greater number of children with RTI in that group to find a reasonable number of M. pneumoniae infections. Using a pre-calculated number of children < 5 years (400) and > 5 years (100), the detectable difference will be 12 % assuming prevalence up to 10% in the < 5 years of age group.

Question 5 (objective 2): do children with M. pneumoniae and a viral or bacterial co-infection have more severe illness?

Analysis: children in group A with a RTI caused by M. pneumoniae will be divided in two groups: children without co-infection, and children with a viral or bacterial co-infection. Severity of disease will be scored according to diagnosis, severity of respiratory distress (days of required oxygen or mechanical ventilation and days of hospitalization. Groups will be compared using Mann-Whitney U test.

Question 6 (objective 3): does genotype of M. pneumoniae influence the rate of asymptomatic carrier state (level of PCR in healthy children, group B), and/or severity of disease?

Analysis: depending on the number of different genotypes of M. pneumoniae found, we will compare these different genotypes to PCR levels using Kruskal-Wallis test. This will be done in the asymptomatic children (healthy M. pneumoniae carrierstate) as well as the children with a M. pneumoniae RTI. Severity of disease will be scored according to severity of respiratory distress (days of required oxygen or mechanical ventilation) and days of hospitalization. This will be compared with different genotype by Kruskal-Wallis test in the symptomatic group (children with M. pneumoniae RTI).

# ETHICAL CONSIDERATIONS

## Regulation statement

The study will be conducted according to the principles of the Declaration of Helsinki (2004) and in accordance with the Medical Research Involving Human Subjects Act (WMO) and the code of conduct as accepted by The Netherlands Association for Paediatric Medicine, June 2003.

## Recruitment and consent

From all eligible patients informed consent will be procured by one of the investigators or a delegated team member (Research Coordinator). Group A patients, visiting the Emergency Department of the Erasmus Medical Centre/Sophia children’s hospital or the HAP, will have a maximum of 24 hours to decide whether or not to participate this study.

In group B parents and children receive an information leaflet on the day they attend the outpatient clinic in preparation of their surgery. A few days later they receive a phone call from a member of the study team to see if there are any questions and to make sure they have all the information they require to be well-informed. If parents and patients decide to participate, the informed consent form is signed. In practice this means healthy controls usually have up to one week to decide about participation in this trial.

The subject or legally acceptable representative should receive a copy of the signed informed consent and any other written information provided to study subjects.

## Objection by minors or incapacitated subjects

Investigators must follow standard procedures to ensure that the minor’s parents or legally acceptable representatives are able to give fully informed written consent. All local laws, rules and regulations regarding informed consent of minors must be followed. For minors, according to local legislation, one or both parents or legally acceptable representative must be informed of the study procedures and must sign the informed consent form approved for the study prior to clinical trial participation. Minors who are ≥ 12 years of age must also give their written assent.

All our subjects are children. Behaviour of all subjects will be assessed at all times during the study procedures. In accordance with the code of conduct accepted by The Netherlands Association for Paediatric Medicine (NVK), June 2001, any behaviour not within bounds of normal behaviour when confronted with situations not encountered in normal day live, will be defined as resistance or an expression of objection. The participation of these subjects will be invalidated, that is, excluded form the study according to law (WMO, art. 4, lid 2).

## Benefits and risks assessment, group relatedness

M. pneumoniae is a major cause of morbidity and mortality in children in both developing countries and Western countries. Knowledge on colonization, and host and bacterial factors contributing to progression to infection is important to optimize diagnosis and treatment, and to prevent transmission of M. pneumoniae.

Obtaining these samples is considered common clinical practice. The risks and burden are negligible.

## Compensation for injury

Not applicable

## Incentives

All participants receive a toy store voucher (€ 5,-) after completing the study procedures.

# ADMINISTRATIVE ASPECTS AND PUBLICATION

## Handling and storage of data and documents

This study will be conducted in accordance with guidelines for Good Clinical practice. All patient data and material will be stored anonymously. Confidentiality will be maintained at all times. All data will be coded with a research number. The key will only be available to members the research team (investigators and research coordinator).

Human material will be kept a maximum of 10 years. Data will be kept for the duration of the study.

## Amendments

Amendments are changes made to the study after a favourable opinion by the accredited METC has been given. All amendments will be notified to the METC that gave a favourable opinion.

Non-substantial amendments will not be notified to the accredited METC and the competent authority, but will be recorded and filed by the investigator.

## Annual progress report

The investigator will submit an annual progress report of the trial to the accredited METC. Information will be provided on the date of inclusion of the first subject, numbers of subjects included and numbers of subjects that have completed the trial, serious adverse events/ serious adverse reactions, other problems, and amendments.

## End of study report

The investigator will notify the accredited METC of the end of the study within a period of 8 weeks. The end of the study is defined as the last patient’s last visit.

In case the study is ended prematurely, the investigator will notify the accredited METC, including the reasons for the premature termination.

Within one year after the end of the study, the investigator/sponsor will submit a final study report with the results of the study, including any publications/abstracts of the study, to the accredited METC.

## Public disclosure and publication policy

Study outcomes and endpoints will be published in an international medical journal. No clinical data will be published.

# Ancillary studies

## Ancillary study 1: Follow-up study to investigate carriage.

### Rationale

Preliminary data from the original MymIC study have shown the presence of *M. pneumoniae* in the respiratory tract of healthy individuals. In addition, the actual bacterial load (determined by real-time PCR) in patients with a RTI seems to be higher than in healthy individuals. These data suggest that asymptomatic colonization with *M. pneumoniae*, defined as a single positive test for *M. pneumoniae* at a certain timepoint, exists. This now leaves room for two options: (1) presence of *M. pneumoniae* in a healthy individual is always associated with an RTI either before or after, reflecting an increasing or declining load, or (2) the presence of *M. pneumoniae* reflects a persistent carriage stage without resulting in infection. At this moment there is no evidence for either of these possibilities and very little is known about *M. pneumoniae* carriage. In this study we will address this issue by performing follow up sampling in both participants with an RTI caused by *M. pneumoniae* and healthy participants with a positive *M. pneumoniae* test (either culture or PCR). The bacterial load and genotype*,* found in these follow-up samples will be related to clinical data in a descriptive manner.

### Objective

To further elucidate the dynamics of asymptomatic colonization of *M. pneumonae* and establishing the value of a positive real-time PCR result.

### Study design

## *Approach*

Children enrolled in the study as described under Aim I in chapter 3, may participate in the follow up study if they tested positive for *M. pneumoniae* by either real time PCR or culture in a sample taken during the first visit. The results of the tests taken during the first visit will be made available to parents and participants about one week prior to the second visit. Subsequently they will be asked to participate in the following protocol

*Definitions*

1. Children with RTI in combination with the presence of *M. pneumoniae,* as detected by either culture or real-time PCR in either the oropharyngeal swab or the nasopharyngeal lavage taken during the first visit, i.e. **group A+** or **group HAP+**. (Note: these children not necessarily suffer from disease caused by infection with *M. pneumoniae*. The gold standard for *M. pneumoniae* infection in this study is serology in combination with standard PCR.)

2. Healthy children tested positive for *M. pneumoniae* by culture or real-time PCR in either the oropharyngeal swab or the nasopharyngeal lavage taken during the first visit, i.e. **group B+**.

*Follow up protocol*

In the follow up protocol participants will be tested for the presence of *M. pneumoniae* in the nasopharynx and oropharynx once a month during a maximum period of one year or until they test negative in both tests (culture and real-time PCR) on two successive occasions. For monthly sample collections, either house-calls will be performed or participants may visit the emergency department of the Erasmus MC – Sophia, whichever is most convenient for participants at that time. If a child, participating in this study, suffers from a RTI during the follow up period, he/she will be evaluated, during daytime, by a physician specialized in pediatric infectious diseases. At all times during the follow up study, parents are free to take him/her to their own general practitioner. For emergencies parents should follow the normal procedures.

*Clinical samples*

Each month a nasopharyngeal lavage and an oropharyngeal swab will be taken and real-time PCR and culture will be performed for *M. pneumoniae* as described previously in chapter 3. Test results will be made available to the participants and their parents every month since these results determine whether the study is continued for each participant individually.

*Clinical data*

Each visit, information about RTI (both participant as well as siblings and parents), use of antibiotics, hospitalization, complications and vaccinations in the previous month will be recorded in a standardized questionnaire.

*Detection of M. pneumoniae*

See Aim I, chapter 3

# *Genotype of M. pneumoniae*

See Aim I, chapter 3. The genotype of *M. pneumonia*e will be determined in each positive sample.

### Study population

## *Population*

Group A+/HAP+: children, aged 0-16 years tested positive for *M. pneumoniae* in group A/HAP by culture or PCR (we expect to find 10% *M. pneumoniae* positive RTI).

Group B+: children, aged 0-16 years tested positive for *M. pneumoniae* in group B by PCR or culture (we expect to find 10% of these children to be positive by PCR).

## *Inclusion criteria*

Group A+/HAP+

Eligible for group A/HAP

Real-time PCR or culture positive for *M. pneumoniae*

Written informed consent for receiving results of the *M. pneumoniae* tests.

Written informed consent for group A/HAP and A+/HAP+

Group B+

Eligible for group B

Real-time PCR or culture positive for *M. pneumoniae*

Written informed consent for receiving results of the *M. pneumoniae* tests.

Written informed consent for group B and B+

## *Exclusion criteria*

Group A+/HAP+

Non-eligible for group A/HAP

Real time PCR or culture negative for *M. pneumoniae*

No written informed consent for receiving results of the *M. pneumoniae* tests.

No written informed consent for group A+/HAP+

Group B+

Not eligible for group B

Real time PCR or culture negative for *M. pneumoniae*

No written informed consent for receiving results of the *M. pneumoniae* tests.

No written informed consent for group B+

## *Sample size calculation*

This study will be merely descriptive. Currently, about 10% of the enrolled children are tested positive by PCR for *M. pneumoniae*. This ancillary study will commence halfway during the original study, so we expect in total an additional 50 children will be tested positive by PCR and will therefore be eligible for this study.

### Study Methods

*Additional visits for follow up*

Group A+/HAP+ and B+ will be asked to return for a maximum of 12 follow-up visits after visit 1. For convenience of participants, house calls will be offered to each participant and their parents and performed by the research team. During these visits a nasopharyngeal lavage and an oropharyngeal swab will be taken and there will be additional short questionnaires.

### Statistics

*Research question(s)*

Does the presence of *M. pneumoniae* in healthy individuals at a certain time point reflect a persistent carriage stage or merely an increasing or declining bacterial load before or after an infection?

*Analysis*

Bacterial load will be determined in samples taken from each visit. These will be used mainly in a descriptive fashion. We hypothesize that a persistent carriage stage does not exist and expect to find clearance of bacteria in all patients eventually. The number of RTI will be scored in each group. The time to clear all bacteria (defined by a negative PCR) will be determined for each individual and means will be compared between each group. The genotype of *M. pneumoniae* will be determined in every sample to compare to ‘time to clear’ and severity of infections.

# REFERENCES

1. Rudan I, Tomaskovic L, Boschi-Pinto C, Campbell H. Global estimate of the incidence of clinical pneumonia among children under five years of age. Bull World Health Organ 2004;82(12):895-903.

2. Michelow IC, Olsen K, Lozano J, Rollins NK, Duffy LB, Ziegler T, et al. Epidemiology and clinical characteristics of community-acquired pneumonia in hospitalized children. Pediatrics 2004;113(4):701-7.

3. Waites KB, Talkington DF. Mycoplasma pneumoniae and its role as a human pathogen. Clin Microbiol Rev 2004;17(4):697-728, table of contents.

4. Principi N, Esposito S, Blasi F, Allegra L. Role of Mycoplasma pneumoniae and Chlamydia pneumoniae in children with community-acquired lower respiratory tract infections. Clin Infect Dis 2001;32(9):1281-9.

5. Johnston SL, Martin RJ. Chlamydophila pneumoniae and Mycoplasma pneumoniae: a role in asthma pathogenesis? Am J Respir Crit Care Med 2005;172(9):1078-89.

6. Taylor-Robinson D, Bebear C. Antibiotic susceptibilities of mycoplasmas and treatment of mycoplasmal infections. J Antimicrob Chemother 1997;40(5):622-30.

7. Waris ME, Toikka P, Saarinen T, Nikkari S, Meurman O, Vainionpaa R, et al. Diagnosis of Mycoplasma pneumoniae pneumonia in children. J Clin Microbiol 1998;36(11):3155-9.

8. Daxboeck F, Krause R, Wenisch C. Laboratory diagnosis of Mycoplasma pneumoniae infection. Clin Microbiol Infect 2003;9(4):263-73.

9. Foy HM. Infections caused by Mycoplasma pneumoniae and possible carrier state in different populations of patients. Clin Infect Dis 1993;17 Suppl 1:S37-46.

10. Gnarpe J, Lundback A, Sundelof B, Gnarpe H. Prevalence of Mycoplasma pneumoniae in subjectively healthy individuals. Scand J Infect Dis 1992;24(2):161-4.

11. Leng Z, Kenny GE, Roberts MC. Evaluation of the detection limits of PCR for identification of Mycoplasma pneumoniae in clinical samples. Mol Cell Probes 1994;8(2):125-30.

12. Dorigo-Zetsma JW, Wilbrink B, van der Nat H, Bartelds AI, Heijnen ML, Dankert J. Results of molecular detection of Mycoplasma pneumoniae among patients with acute respiratory infection and in their household contacts reveals children as human reservoirs. J Infect Dis 2001;183(4):675-8.

13. Principi N, Esposito S. Emerging role of Mycoplasma pneumoniae and Chlamydia pneumoniae in paediatric respiratory-tract infections. Lancet Infect Dis 2001;1(5):334-44.

14. Layani-Milon MP, Gras I, Valette M, Luciani J, Stagnara J, Aymard M, et al. Incidence of upper respiratory tract Mycoplasma pneumoniae infections among outpatients in Rhone-Alpes, France, during five successive winter periods. J Clin Microbiol 1999;37(6):1721-6.

15. Plouffe JF. Importance of atypical pathogens of community-acquired pneumonia. Clin Infect Dis 2000;31 Suppl 2:S35-9.

16. Toikka P, Juven T, Virkki R, Leinonen M, Mertsola J, Ruuskanen O. Streptococcus pneumoniae and Mycoplasma pneumoniae coinfection in community acquired pneumonia. Arch Dis Child 2000;83(5):413-4.

17. Baseman JB. The cytadhesins of Mycoplasma pneumoniae and M. genitalium. Subcell Biochem 1993;20:243-59.

18. Leith DK, Trevino LB, Tully JG, Senterfit LB, Baseman JB. Host discrimination of Mycoplasma pneumoniae proteinaceous immunogens. J Exp Med 1983;157(2):502-14.

19. Su CJ, Chavoya A, Baseman JB. Regions of Mycoplasma pneumoniae cytadhesin P1 structural gene exist as multiple copies. Infect Immun 1988;56(12):3157-61.

20. Wenzel R, Herrmann R. Repetitive DNA sequences in Mycoplasma pneumoniae. Nucleic Acids Res 1988;16(17):8337-50.

21. Kenri T, Taniguchi R, Sasaki Y, Okazaki N, Narita M, Izumikawa K, et al. Identification of a new variable sequence in the P1 cytadhesin gene of Mycoplasma pneumoniae: evidence for the generation of antigenic variation by DNA recombination between repetitive sequences. Infect Immun 1999;67(9):4557-62.

# APPENDIX

## Flowchart FUP study


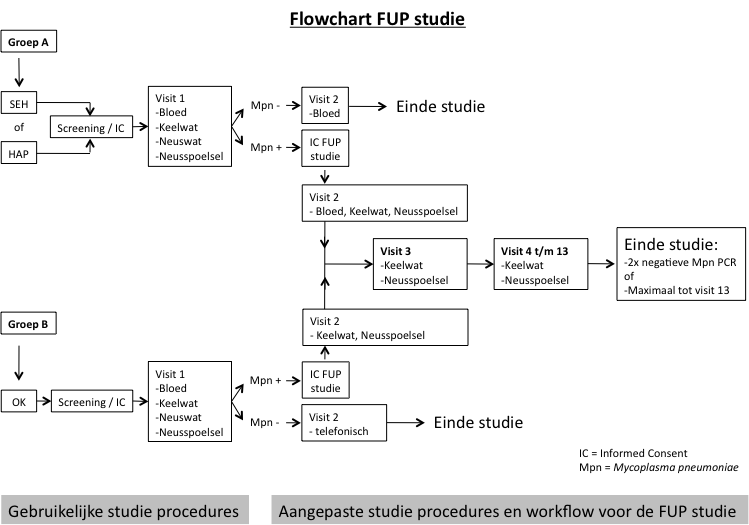

Supplement: Text S1 — Study protocol. (DOC) [file pmed.1001444.s005.doc]
